# p20BAP31 induces cell apoptosis via both AIF caspase-independent and the ROS/JNK mitochondrial pathway in colorectal cancer

**DOI:** 10.1186/s11658-023-00434-z

**Published:** 2023-03-28

**Authors:** Xiaohan Jiang, Guoxun Li, Benzhi Zhu, Jingnan Zang, Tian Lan, Rui Jiang, Bing Wang

**Affiliations:** College of Life and Health Science, Northeastern University, 195 Chuangxin Road, Hunnan District, Shenyang, Liaoning Province China

**Keywords:** p20BAP31, Apoptosis, AIF, ROS/JNK pathway, Colorectal cancer

## Abstract

**Background:**

During cell apoptosis, the C-terminus of BAP31 is cleaved by caspase-8 and generates p20BAP31, which has been shown to induce an apoptotic pathway between the endoplasmic reticulum (ER) and mitochondria. However, the underlying mechanisms of p20BAP31 in cell apoptosis remains unclear.

**Methods:**

We compared the effects of p20BAP31 on cell apoptosis in six cell lines and selected the most sensitive cells. Functional experiments were conducted, including Cell Counting Kit 8 (CCK-8), reactive oxygen species (ROS), and mitochondrial membrane potential (MMP) assay. Then, cell cycle and apoptosis were investigated by flow cytometry and verified by immunoblotting. Next, NOX inhibitors (ML171 and apocynin), ROS scavenger (NAC), JNK inhibitor (SP600125), and caspase inhibitor (Z-VAD-FMK) were used to further investigate the underlying mechanisms of p20BAP31 on cell apoptosis. Finally, apoptosis-inducing factor (AIF) translocation from the mitochondria to the nuclei was verified by immunoblotting and immunofluorescence assay.

**Results:**

We found that overexpression of p20BAP31 indeed induced apoptosis and had a much greater sensitivity in HCT116 cells. Furthermore, the overexpression of p20BAP31 inhibited cell proliferation by causing S phase arrest. Further study revealed that p20BAP31 reduced MMP, with a significant increase in ROS levels, accompanied by the activation of the MAPK signaling pathway. Importantly, the mechanistic investigation indicated that p20BAP31 induces mitochondrial-dependent apoptosis by activating the ROS/JNK signaling pathway and induces caspase-independent apoptosis by promoting the nuclear translocation of AIF.

**Conclusions:**

p20BAP31 induced cell apoptosis via both the ROS/JNK mitochondrial pathway and AIF caspase-independent pathway. Compared with antitumor drugs that are susceptible to drug resistance, p20BAP31 has unique advantages for tumor therapy.

**Supplementary Information:**

The online version contains supplementary material available at 10.1186/s11658-023-00434-z.

## Introduction

B-cell receptor associated protein 31 (BAP31), a polytopic integral endoplasmic reticulum (ER) membrane protein that is part of a complex that contains Bcl-2/Bcl-X_L_ and procaspase 8, has been implicated in the ER sorting of diverse client membrane proteins [1–4]. BAP31 has been reported to transport transmembrane proteins, such as MHC-1, CD11b/CD18, and cytochrome 450, from the ER to other cellular components [4–7]. Our previous study reported that BAP31 associates with the N-terminus of CFTRΔF508 and promotes its retro-translocation from the ER and its degradation by the cytoplasmic 26S proteasome system [8]. Our recent studies demonstrated that BAP31 is involved in T-cell activation [9], hepatic lipid accumulation, insulin resistance [10], and could combine with p27^kip1^ and regulate its proteasome degradation [11]. Furthermore, BAP31 depletion hindered human embryonic stem cell (hESC) proliferation by arresting cells in the G0/G1 phase and inducing caspase-independent cell death [12]. Recent studies have shown that BAP31 communicates with the ER and mitochondria through contact sites to regulate mitochondrial functions and autophagy [13] and is also considered a novel tumor suppression factor involved in metabolic stress, inducing cell death via the ER stress response [14].

The cytosolic domain of BAP31 contains two identical caspase recognition sites (AAVD.G). After activation of cell surface death receptors, BAP31 is cleaved by caspase-8 and generates a membrane-embedded fragment called p20BAP31, which has been shown to direct proapoptotic signals between the ER and mitochondria when expressed ectopically [3, 15]. Previous studies have shown that p20BAP31 stimulates ER Ca^2+^ release, resulting in the activation of Drp1-dependent fission of mitochondria and enhancing the release of cytochrome c (cyt.c) [16]. It is worth mentioning that p20BAP31 was shown to induce apoptosis even in the absence of endogenous BAP31 [16], which can exert dominant-negative (DN) interference with the protein transport functions of full-length BAP31 [5, 8, 17]. Furthermore, a previous study identified that ER localized Bcl-2 protects against a Bax/Bak-independent paraptosis-like cell death pathway initiated by p20BAP31 [18]. In conclusion, p20BAP31 not only stimulates ER Ca^2+^ release, mitochondrial fission, and release of cyt.c to induce cell apoptosis [16], but also initiates a paraptosis-like cell death pathway [18]. However, the mechanism of p20BAP31-induced cell apoptosis is not fully understood, and whether p20BAP31 can trigger other types of cell death remains unclear.

Programmed cell death (PCD) plays a crucial role in many biological processes [19]. Among the different forms of programmed cell death, cell apoptosis is the most common and best studied and is divided into the extrinsic and intrinsic apoptotic pathways [20, 21]. Both pathways use caspases to carry out apoptosis through the cleavage of hundreds of proteins [22]. In the caspase-dependent pathway, proapoptotic proteins of the Bcl-2 family cause changes in mitochondrial membrane permeability [23–25]. Previous studies have shown that p20BAP31 stimulates mitochondrial fission [16]; however, the change in mitochondrial membrane potential has not been documented. In the caspase-independent pathway, apoptosis-inducing factor (AIF) was discovered as the first protein that regulates caspase-independent apoptosis, which is released from mitochondria and then translocates to the nucleus, where it contributes to chromatin condensation and DNA degradation [26–29]. In previous studies, p20BAP31 has been shown to induce the release of cyt.c from mitochondria [16], but whether AIF can be released from mitochondria is still unknown.

Reactive oxygen species (ROS) are produced through a variety of intracellular and extracellular activities. Excessive cellular levels of ROS cause damage to proteins, nucleic acids, lipids, membranes, and organelles, leading to the activation of cell death processes such as apoptosis [30, 31]. ROS generation is regulated primarily by NADPH oxidase (NOX) enzymes, which predominantly release hydrogen peroxide (H_2_O_2_) and superoxide (O_2_^−^); four NADPH oxidases (NOX1, NOX2, NOX4, and NOX5) play a prominent role in homeostasis and disease [32]. Furthermore, ROS-induced oxidative stress activates the mitogen-activated protein kinase (MAPK) signaling pathway [33]. Therefore, we explored the effect of p20BAP31 on ROS, the source of p20BAP31-induced ROS, and further explored whether it can activate the MAPK signaling pathway. The MAPK signaling pathway is the key to triggering a variety of cellular responses, such as proliferation, apoptosis, differentiation, and survival. MAPKs, including ERK, JNK, and p38, play a critical role in chemical-triggered cell cycle arrest and apoptotic processes [34]. ERK is generally involved in proliferation and growth regulation. JNK activation is associated with transformation in many oncogene and growth factor-mediated pathways. Activation of p38 can promote cell proliferation or induce cell cycle arrest [33]. Consequently, it is necessary to explore the effects of p20BAP31 on cell cycle progression.

Previous studies have reported that p20BAP31 triggers at least two different proapoptotic events: cristae remodeling, which sensitizes mitochondria to apoptosis, and opening of the protein tyrosine phosphatome (PTP), which alone is sufficient to trigger apoptosis. Another interesting point is that there appears to be a BAP31-dependent setup in place that enables the ER to integrate/amplify apoptotic signals originating in mitochondria [35]. These reports suggest that p20BAP31 can induce an apoptotic pathway between the ER and mitochondria, but how p20BAP31-induced apoptosis occurs, and which apoptosis-related pathways are involved, remains poorly understood. In this study, we found that p20BAP31 induced cell apoptosis in various cells, when the transduction efficiency of p20BAP31 in these cells was similar, HCT116 cells showed more sensitivity. Therefore, HCT116 cells were selected as model cells for further study. The overexpression of p20BAP31 promoted the production of ROS and activated the MAPK signaling pathway, and mitochondrial membrane potential (MMP) was also significantly decreased. Further investigation confirmed that p20BAP31 induced mitochondrial-dependent apoptosis by activating the ROS/JNK signaling pathway. In addition, we found that p20BAP31 induced cell cycle arrest in the S phase, different from knockdown of BAP31, which induced cell arrest in the G0/G1 phase [12]. We were also surprised to find that p20BAP31 induced AIF-mediated apoptosis, which was independent of caspase. In combination with previously published data that p20BAP31 initiates a paraptosis-like cell death pathway that does not depend on Bax/Bak [18], we concluded that p20BAP31 induces apoptosis through different pathways. Therefore, when certain apoptosis signaling pathways are inhibited, cell apoptosis can still occur through other pathways. This property of inducing cell apoptosis through multiple pathways makes p20BAP31 potentially valuable in the research of antitumor therapy.

## Materials and methods

### Reagents and antibodies

*N*-Acetyl-l-cysteine (NAC) and Z-VAD-FMK (caspase inhibitor) were purchased from Beyotime (Shanghai, China). SP600125 (JNK inhibitor) was purchased from Sigma‒Aldrich (St. Louis, MO, USA). ML171 (NOX1 inhibitor) and apocynin (NOX2 inhibitor) were purchased from ApexBio (Shanghai, China). Primary antibodies against BAP31, Bcl-2, Bax, caspase-3, cytochrome c, ERK, phospho-ERK, JNK, phospho-JNK, p38, phospho-p38, cyclin E1, CDK2, and p21 were purchased from Cell Signaling Technology (Dancers, MA). Primary antibody against E2F1 and caspase-9 was purchased from Wanleibio (Shenyang, China). Primary antibodies against HA, β-actin, PARP, cyclin A2, and AIF were purchased from Beyotime (Shanghai, China). Primary antibodies against TOM20 and histone H3 were purchased from Absin Bioscience Inc. (Shanghai, China).

### Cell culture

The human colorectal carcinoma cell line HCT116 (cat. no. TCHu99), the human non-small cell lung cancer cell line A549 (cat. no. TCHu150), the human bladder transitional cell carcinoma cell line UM-UC-3 (cat. no. TCHu217), the human hepatocarcinoma cell line HepG2 (cat. no. TCHu72), and the human embryonic kidney cell line 293 T (cat. no. GNHu17) were obtained from the cell bank of the Chinese Academy of Sciences (Shanghai, China). The human gastric cancer cell line BGC823 (cat. no. LM-80054) was purchased from LMAI Bio (Shanghai, China). All the above cell lines were cultured in DMEM (Gibco, NY, USA) supplemented with 10% fetal bovine serum (FBS), 1% l-glutamine and 1% penicillin‒streptomycin (Gibco) at 37 °C in a 5% CO_2_ incubator (Thermo Fisher Scientific, Waltham, MA, USA). All cell lines were confirmed to be mycoplasma free by the Hoechst DNA stain (indirect) method.

### Plasmid construction and transfection

The pcDNA3.1(-) plasmid (Mock) was obtained from Thermo Fisher Scientific (Waltham, MA, USA), and the pEGFP-N1 vector was obtained from TaKaRa (Beijing, China). Standard polymerase chain reaction (PCR) techniques were used to generate cDNA encoding p20BAP31 (aa 1–164 of human BAP31) with a COOH-terminal HA tag that was cloned into the *EcoRI* and *BamHI* sites of the pcDNA3.1(-) and pEGFP-N1 vectors (PCR primer sequences are shown in Additional file 5: Table S1). Cells were transiently transfected using Lipo8000 transfection reagent (Beyotime, Shanghai, China). Briefly, 3 × 10^5^ cells were plated in 6-well plates the day before transfection. The plasmid to Lipofectamine ratio was 1:1.5. Two micrograms of plasmids were mixed in 125 μL of Opti-MEM, and then 3 μL Lipo8000 transfection reagent was added. The solution was mixed gently and incubated at room temperature for 15 min. Then, the solutions were added to the plate dropwise. Six hours later, the medium was replaced with fresh medium, and the cells were harvested at appropriate times.

### Cell proliferation assay

The mock and p20BAP31 groups were transiently transfected, as described above, the day after seeding in 96-well plates (3000 cells per well). The control group was directly seeded in 96-well plates (3000 cells per well), with five replicate wells per group, and the plate was incubated at 37 °C in a 5% CO_2_ incubator. After each treatment period (0, 24, 48, and 72 h), 10 μL of CCK-8 (Cell Counting Kit 8) solution (Beyotime, Shanghai, China) was added to each well, and the plate was incubated for 2 h. The optical density (OD) of the samples was measured by a Synergy H1 microplate reader (Biotek, USA) at a wavelength of 450 nm.

### Cell apoptosis analysis

Cells (3 × 10^5^ per well) were seeded in 6-well plates, harvested after 48 h of transfection, washed repeatedly with cold phosphate-buffered saline (PBS), and resuspended in 195 μL of binding buffer. Annexin V-FITC reagent (4 μL) and propidium iodide (PI) (8 μL, Beyotime) were added to the samples and mixed gently. After 15 min of incubation in the dark at room temperature, the stained cells were analyzed immediately using a FACScan flow cytometer (BD Biosciences, San Jose, CA, USA) and analyzed with the annexin V-FITC/PI apoptosis method.

### Western blot analysis

Cells (3 × 10^5^ per well) were seeded in 6-well plates and transfected for 48 h, cells were lysed in radioimmunoprecipitation assay (RIPA) buffer (Beyotime, Shanghai, China) containing 1% protease inhibitor cocktail and phenylmethylsulfonyl fluoride (PMSF) (1 mM, Sigma‒Aldrich, St. Louis, MO, USA) for 30 min. After centrifugation at 12,000 × *g* for 15 min, the supernatant was collected. Mitochondria proteins were isolated and lysed using a Cell Mitochondria Isolation Kit (Beyotime). The intranuclear and extranuclear proteins were extracted using Nuclear and Cytoplasmic Protein Extraction Kit (Beyotime) according to the manufacturer’s protocol. The protein concentration was determined by the bicinchoninic acid assay (BCA) method. BCA kits were purchased from Beyotime. After determination of the protein concentrations, the lysates were mixed with the 5 × loading buffer and denatured by boiling for 10 min. The lysates with equal amounts of protein were separated by 10–15% sodium dodecyl-sulfate polyacrylamide gel electrophoresis (SDS‒PAGE), and then transferred to polyvinylidene difluoride (PVDF) membrane (Merk Millipore, Darmstadt, Germany). After blocking with 5% skimmed milk at room temperature for 0.5 h, the PVDF membrane was incubated with the corresponding antibodies at 4 °C overnight and then with horseradish peroxidase (HRP)-conjugated secondary antibodies at room temperature for 1 h. Finally, the protein bands were visualized with a Bio-Rad ChemiDoc imaging system using an ECL detection kit (Thermo Fisher Scientific, Waltham, MA, USA).

### Hoechst staining

HCT116 cells (5 × 10^4^ per well) were seeded in 24-well plates and transfected for 48 h. Afterward, the cells were washed three times with phosphate-buffered saline (PBS) and incubated with Hoechst 33342 (Beyotime, 2 μg/mL in medium) at room temperature for 5 min in the dark. The nuclear images were observed with a DMI3000B fluorescence microscope (Leica, Germany), and the changes in nuclear morphology were evaluated and photographed.

### Colony forming assay

HCT116 cells were seeded into six-well plates at a density of 2000 cells per well and transfected with the plasmid. All conditions were performed in triplicate using untreated cells as a reference control. Cells were cultivated for 7 days, at which point they were fixed with 4% formaldehyde for 30 min and stained with 0.1% crystal violet for 15 min. Finally, the plates were washed with water and air-dried at room temperature. The colony counts were determined using ImageJ software (v. 1.8.0) (NIH, Bethesda, MD, USA).

### Mitochondrial membrane potential (MMP) assay

MMP was examined using a JC-1 mitochondrial membrane potential assay kit (Beyotime, Shanghai, China) according to the manufacturer’s instructions. Briefly, HCT116 cells (3 × 10^5^ per well) were plated in six-well plates. After transfection with plasmid for 48 h, cells were collected and mixed with 1 mL of 1 × JC-1 working solution at 37 °C in the dark for 20 min. Subsequently, the cells were centrifuged (1000 rpm, 5 min) and washed twice with 1 × JC-1 staining buffer. The collected cells were resuspended in 500 μL of JC-1 staining buffer and immediately analyzed by FACScan flow cytometry (BD Biosciences, San Jose, CA, USA).

MMP was also examined by fluorescence microscopy. HCT116 cells (5 × 10^4^ per well) were plated in 24-well plates. After transfection with plasmid for 48 h, the cells were washed once with PBS. Afterward, 0.5 mL of the cell culture solution and 0.5 mL of JC-1 working solution were successively added, thoroughly mixed, and incubated at 37 °C for 20 min in the dark. The cells were washed twice with 1 × JC-1 staining buffer, and fresh culture medium was added. The images were observed with a DMI3000B fluorescence microscope (Leica, Germany).

### Measurement of intracellular ROS levels

ROS levels were quantified in accordance with the protocol of the Reactive Oxygen Species Assay Kit (Beyotime, Shanghai, China). The Reactive Oxygen Species Assay Kit allows for the quantitative measurement of ROS, namely superoxide radicals in cells undergoing oxidative stress. HCT116 cells (3 × 10^5^ per well) were plated in 6-well plates. After transfection with plasmid for 48 h, the cells were stained with diacetyldichlorofluorescein (DCFH-DA 10 μM) at 37 °C for 20 min in the dark and then washed with serum-free Dulbecco's Modified Eagle Medium (DMEM) three times. Finally, the cells were collected for ROS analysis using a FACScan flow cytometer (BD Biosciences, San Jose, CA, USA).

In addition, ROS levels were also examined by fluorescence microscopy. HCT116 cells (5 × 10^4^ per well) were seeded into 24-well plates. After transfection with plasmid for 48 h, the cells were stained with DCFH-DA (10 μM) at 37 °C for 20 min in the dark and then washed with serum-free DMEM three times. Finally, the images were observed with a DMI3000B fluorescence microscope (Leica, Germany).

### Cell cycle analysis

For the cell cycle distribution analysis, HCT116 cells (3 × 10^5^ per well) were plated in six-well plates. After transfection with plasmid for 48 h, the cells were collected with growth medium, centrifuged, washed with cold PBS, and fixed with 70% ice-cold ethanol overnight at –20 °C. The next day, the cells were washed twice with cold PBS, and gently resuspended in 500 μL of PI working fluid (staining buffer: PI (20 ×): RNAase (50 ×) ratio is 100: 5: 2) (Meilun Biotechnology Co. Ltd., Dalian, China) and incubated at 37 °C for 30 min in the dark. Cell cycle analysis was performed using a FACScan flow cytometer (BD Biosciences, San Jose, CA, USA).

### Immunofluorescence assay

A total of 5 × 10^4^ cells were plated in 24-well plates. After 48 h of transfection with plasmid, the cells were fixed in 4% paraformaldehyde for 30 min, permeabilized with 0.2% Triton X-100 for 30 min, blocked with 1% BSA-PBS for 30 min at room temperature, and then incubated with anti-AIF antibody (1:100 dilution) at 4 °C overnight. The fluorescent secondary antibodies were incubated with the cells at room temperature for 2 h in the dark. The nuclei were stained with 4′,6-diamidino-2-phenylindole (DAPI, 300 nM in PBS) for 5 min, and the images were captured using a DMI3000B fluorescence microscope (Leica, Germany).

### Statistical analysis

All results are presented as mean ± standard error (SE) from at least three independent experiments. Curve fittings, data, and statistical analysis were carried out by the GraphPad Prism program (Prism 6, San Diego, CA, USA). Differences between groups were assessed by one-way analysis of variance (ANOVA). For all analyses, *P* values represent 2-sided tests of statistical significance effects. *P* values < 0.05 were considered statistically significant. SPSS version 13.0 (IBM, NY, USA) was used to calculate the differences. Nonlinear regression was fitted to the data points using the GraphPad Prism program.

## Results

### p20BAP31 induced apoptosis and inhibited proliferation

**Fig. 1:**
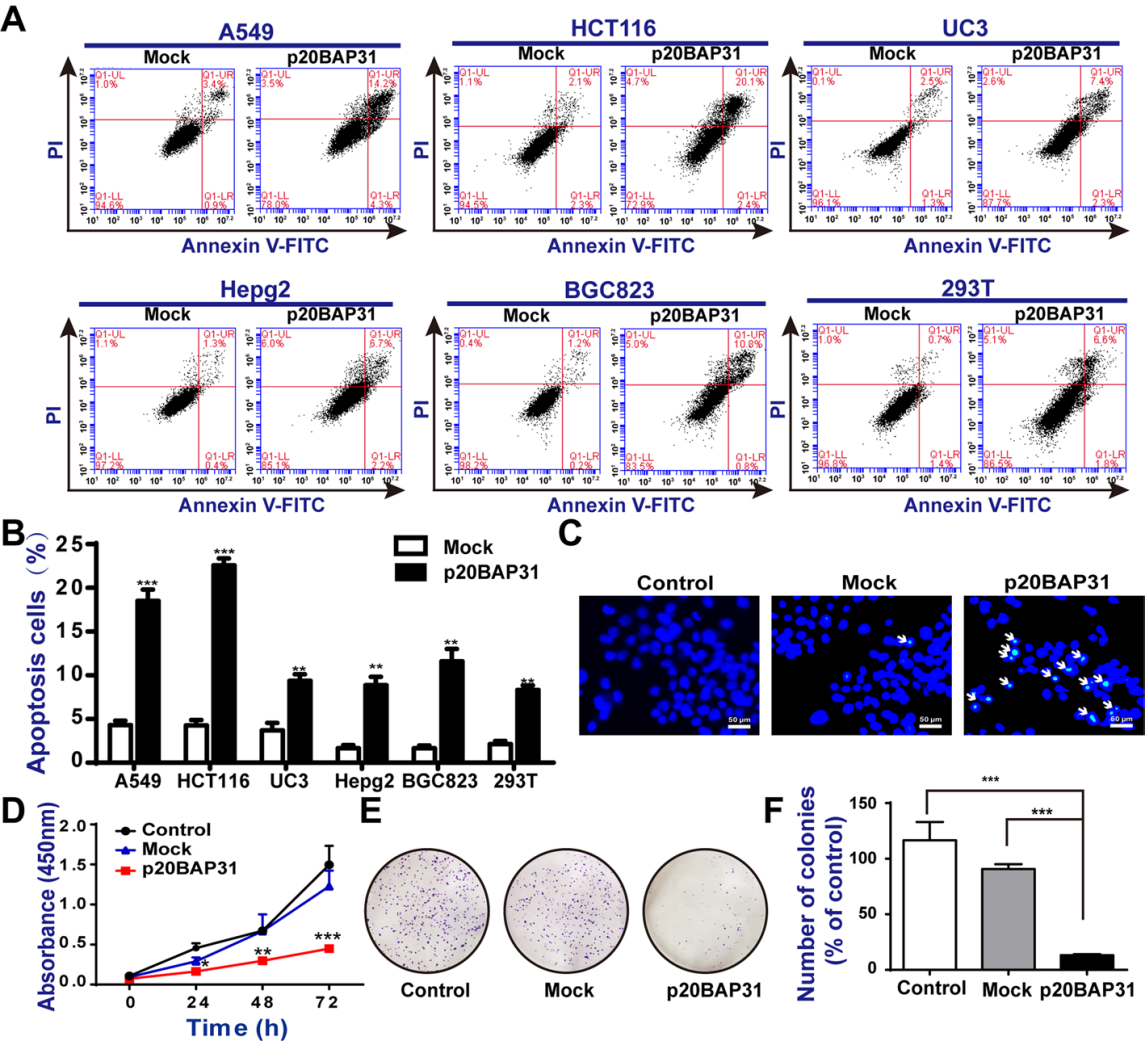
p20BAP31 induced cell apoptosis and inhibited cell proliferation. **A** Cells were transfected with p20BAP31 for 48 h, and apoptosis was assessed by flow cytometry. **B** Quantitative analysis of apoptosis rates by flow cytometry. **C** HCT116 cells were transfected with p20BAP31 for 48 h. Cell nuclei were observed by fluorescence microscopy. Typical apoptosis morphological changes were shown in treated cells, including chromatin condensation and DNA fragmentation. **D** HCT116 cells were transfected with p20BAP31 for 0, 24, 48, and 72 h. The absorbance value was determined using the CCK-8 assay, the significant difference was compared between the mock group and the p20BAP31 group. **E** Colony formation was assayed on a 6-well plate for 7 days, and then, the HCT116 cells were fixed with 4% formaldehyde for 30 min and stained with 0.1% crystal violet for 15 min. Representative images, which are shown at × 1 magnification **E**, and the bar graph of the quantitation **F** indicate the colony formation of HCT116 cells. The data are shown as the mean ± SD of three independent experiments. *P < 0.05, **P < 0.01, ***P < 0.001

To investigate the effects of p20BAP31 on cell apoptosis, five cancer cell lines (A549, HCT116, UC3, HepG2, and BGC823) and human embryonic kidney cells (HEK-293T) were transfected with HA-tagged p20BAP31 for 48 h. As shown in Additional file 1: Figs. S1A, S1B, the protein expression levels of p20BAP31 in these cells were similar, except for the 293T cells, as the cleavage product of BAP31, p20BAP31, can also be detected by BAP31 antibody. In addition, the transfection efficiency of p20BAP31-EGFP in these cells was further verified, as shown in the Additional file 1: Fig. S1C. Then, p20BAP31-induced apoptosis in various cells was analyzed by flow cytometry (Fig. 1A). The results showed that the proportions of total apoptotic cells (including early and late apoptotic cells) were markedly increased after transfection with p20BAP31 in all the tested cells (Fig. 1B). The apoptosis proportion induced by p20BAP31 in the HCT116 cells was highest among all the tested cells, approximately 22.5%. Therefore, HCT116 cells were chosen for the following research. Interestingly, the protein expression level of p20BAP31 was higher in 293T cells than in HCT116 cells; however, the apoptosis proportion in 293T cells was only 8.4%, which may indicate that the sensitivity of apoptosis activity induced by p20BAP31 was different in cancer cells and normal cells. In addition, we observed the effect of p20BAP31-induced apoptosis on cell morphology. The cells were stained with Hoechst 33342, which showed that the cells transfected with p20BAP31 exerted the typical features of apoptosis, including chromatin shrinkage, membrane blebbing, and the formation of apoptotic bodies (Fig. 1C).

To analyze the effect of p20BAP31 on cell proliferation, we initially determined the effects of p20BAP31 on growth by CCK-8 assay, as shown in Fig. 1D. Compared with the control and mock groups, overexpression of p20BAP31 significantly suppressed the proliferation of HCT116 cells in a time-dependent manner. These results were further confirmed by a colony formation assay (Fig. 1E). Cells transfected with p20BAP31 revealed a significant decrease in colony number compared with the control and mock groups (Fig. 1F). These results verified that p20BAP31 could inhibit cell proliferation and induce cell apoptosis, and have a much greater sensitivity in HCT116 cells.

### p20BAP31 induced cell cycle arrest at S phase in HCT116 cells

**Fig. 2:**
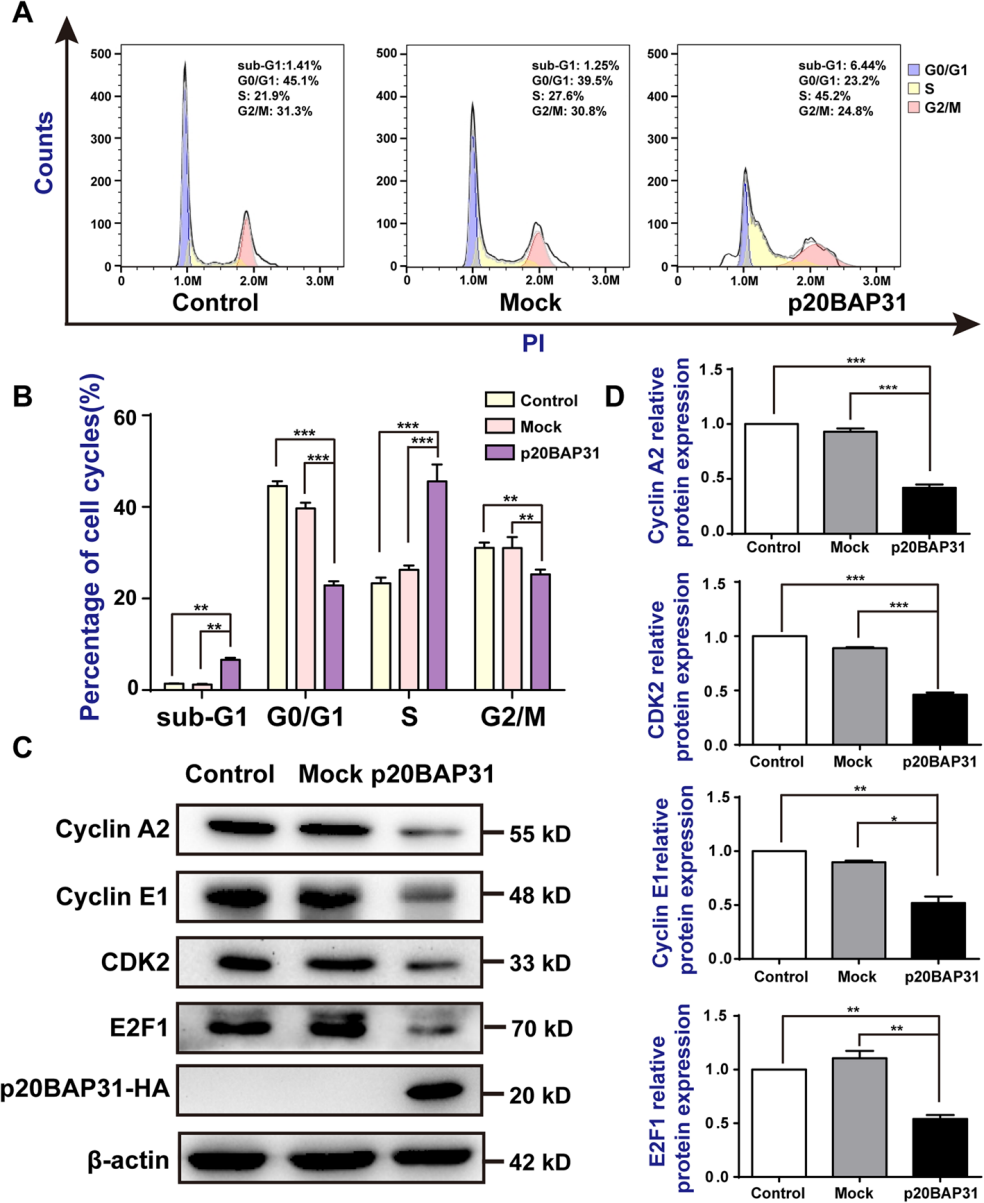
p20BAP31 induced cell cycle arrest at S phase.** A** Flow cytometry was used to determine the cell cycle distribution of HCT116 cells transfected with p20BAP31 for 48 h. **B** Statistical analysis of the cell cycle distribution of HCT116 cells. **C, D** The expression levels of cyclin A2, cyclin E1, and CDK2 in HCT116 cells were measured by western blot analysis after transfection with p20BAP31 for 48 h. *P < 0.05, **P < 0.01, ***P < 0.001

We first determined the optimal transfection time, and fluorescence microscopy was used to record the transfection efficiency and cell morphology changes at different time points after transfection of the p20BAP31-EGFP plasmid (Additional file 2: Fig. S2). The results showed that p20BAP31 was expressed from 12 h, the expression levels remained basically stable for over 48 h, and the cells began to show obvious apoptotic morphology at 48 h. Therefore, we chose 48 h as the optimal transfection time for subsequent experiments. To further explore the effect of p20BAP31 on the proliferation of HCT116 cells, flow cytometry of propidium iodide staining was performed to analyze the cell cycle distribution. The results indicated that the cells transfected with p20BAP31 showed a significant increase in the number of cells in the S phase (Fig. 2A, B). A similar result was obtained after transfected with p20BAP31 for 36 h (Additional file 3: Fig. S3C, S3D), which was not obvious for 24 h (Additional file 3: Fig. S3A, S3B). Specifically, we found that p20BAP31 could increase the relative proportion of sub-G1 (Fig. 2A, B), which is a hallmark of apoptosis [36].

Cyclins and cyclin-dependent kinases (CDKs) play an important role in regulating the cell cycle [37]. In the late G1 stage, the complex of cyclin E and CDK2 is formed to induce a phase transition between G1 and S [38]. In the S phase, cyclin A binds CDK2 to accelerate the cell cycle. To confirm this change, we performed western blot analysis to examine the levels of cell cycle-related proteins. The results showed that p20BAP31 significantly decreased the expression of cyclin A2, cyclin E1, and CDK2 in HCT116 cells (Fig. 2C, D). Previous studies have identified cyclin A2 and cyclin E1 as transcriptional target genes of E2F transcription factor 1 (E2F1) [39]. E2F1 regulated the activity of cyclin A2-CDK2 and cyclin E1-CDK2 complexes, which play crucial roles in the G1-S transition [40, 41]. Therefore, we detected the change in E2F1 after transfection with p20BAP31 in HCT116 cells. The results showed that p20BAP31 effectively reduced the levels of E2F1 (Fig. 2C, D). These data indicate that p20BAP31 inhibits the proliferation of HCT116 cells via E2F1-regulated S phase cell cycle arrest.

### p20BAP31 reduced MMP with a significant increase in ROS levels in HCT116 cells

**Fig. 3:**
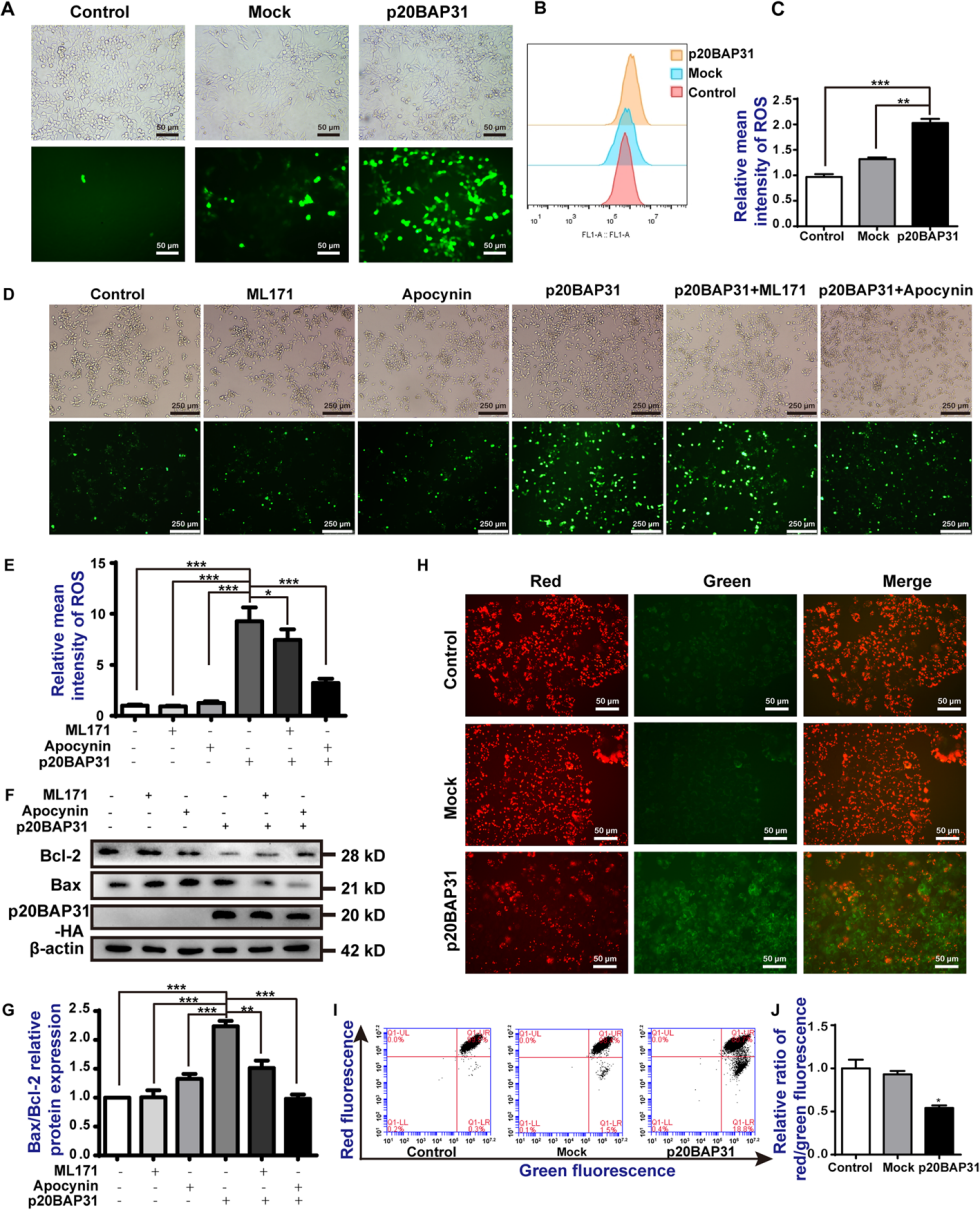
p20BAP31 reduced MMP with a significant increase in ROS levels in HCT116 cells. **A** After transfection with p20BAP31 for 48 h, the cells were collected, and DCFH-DA fluorescence was observed by fluorescence microscopy. **B** Flow cytometry was used to determine the alteration of ROS content in HCT116 cells. **C** Quantitative analysis of ROS in HCT116 cells. **D** HCT116 cells were pretreated with 10 μM ML171 and 10 μM Apocynin for 1 h, and then transfected with p20BAP31 for 48 h. DCFH-DA fluorescence was observed by fluorescence microscopy. **E** Quantitative analysis of the DCFH-DA fluorescence intensity in HCT116 cells. **F** HCT116 cells were pretreated with 10 μM ML171 and 10 μM apocynin for 1 h and then transfected with p20BAP31 for 48 h. Western blotting was used to detect Bcl-2 and Bax levels. **G** The relative quantification of the protein levels was analyzed by ImageJ software.** H** The JC-1 fluorescence distribution in HCT116 cells transfected with p20BAP31 for 48 h was observed under a fluorescence microscope. **I** Changes in MMP levels in HCT116 cells were detected by flow cytometry analysis. **J** Quantitative analysis of the mitochondrial membrane potential of HCT116 cells. *P < 0.05, **P < 0.01 and ***P < 0.001

It has been reported that the mitochondria dysfunction is responsible for apoptosis [42, 43]. To explore the mechanism of p20BAP31-induced apoptosis, we first tested whether p20BAP31 could induce mitochondrial damage. In the process of mitochondrial apoptosis, intracellular ROS is highly induced [44, 45]. Therefore, we detected the production of ROS, as illustrated in Fig. 3A, and compared with the control and mock groups, the green fluorescence intensity of HCT116 cells transfected with p20BAP31 was significantly enhanced. Likewise, the results of flow cytometry showed the accumulation of ROS (Fig. 3B, C). To further examine the source of ROS induced by p20BAP31, we used ML171 (a NOX1 inhibitor), and apocynin (a NOX2 inhibitor). We found that apocynin significantly attenuated p20BAP31-induced ROS production (Fig. 3D, E). Western blot analysis also showed that apocynin inhibited p20BAP31-induced activation of apoptosis-related proteins to a certain extent (Fig. 3F, G). These results suggest that ROS production plays an important role in p20BAP31-induced apoptosis in HCT116 cells, and NOX2 may be the main ROS source induced by p20BAP31.

Mitochondrial membrane potential (MMP) is one of the important indicators for maintaining normal mitochondrial configuration and function [46]. Decreased MMP is one of the important factors leading to apoptosis [47]. To further investigate the effect of p20BAP31 on mitochondrial function, we investigated the ability of p20BAP31 to induce the loss of MMP. The JC-1 staining results revealed that MMP was obviously decreased in HCT116 cells transfected with p20BAP31 (Fig. 3H–J). The intensity of green fluorescence was significantly increased and the intensity of red fluorescence was decreased in the cells transfected with p20BAP31 (Fig. 3H). The evident red-to-green shift in fluorescence suggested a shift from JC-1 dimers to JC-1 monomers. The results of flow cytometry revealed that the ratio of red and green fluorescence decrease by approximately 50% in the cells transfected with p20BAP31 when compared with the control and mock groups (Fig. 3J). Meanwhile, the MMP levels drastically dropped to 80.7% in HCT116 cells transfected with p20BAP31 (Fig. 3I). These observations suggested that p20BAP31 induced depolarization of the mitochondrial membrane, which could be linked to the induction of apoptosis in HCT116 cells.

### Effects of p20BAP31 on apoptosis-related proteins in HCT116 cells

**Fig. 4:**
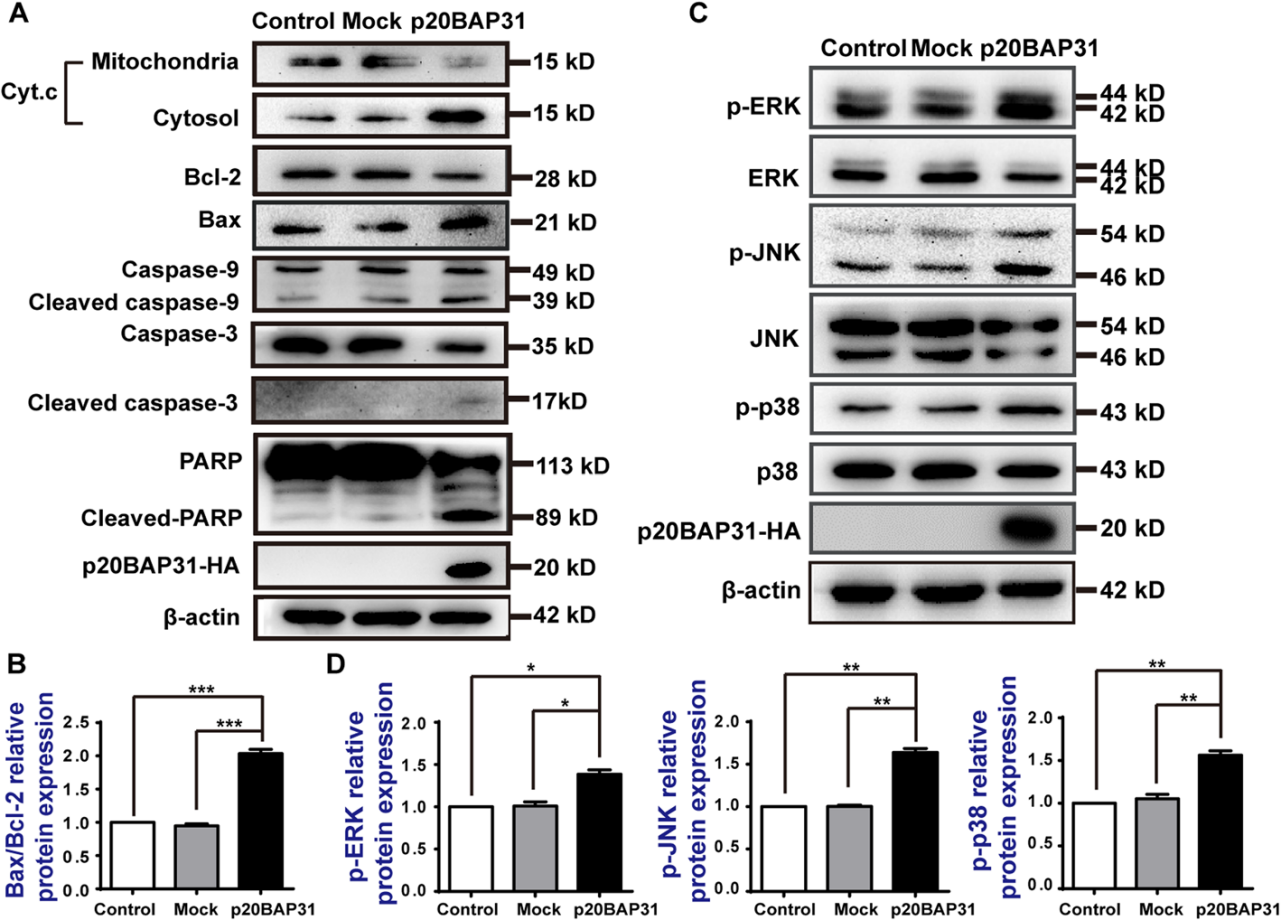
p20BAP31 induces mitochondrial apoptosis by activating the MAPK signaling pathway. **A** The expression levels of Bcl-2, Bax, caspase-3, cleaved caspase-3, PARP, cleaved PARP, caspase-9, cleaved caspase-9, and cyt.c proteins in HCT116 cells were determined by western blot after transfection with p20BAP31 for 48 h. **B** Relative quantity analysis of Bax/Bcl-2 in HCT116 cells. **C** Western blotting was used to detect p-ERK, ERK, p-JNK, JNK, p-p38, and p38 levels in HCT116 cells transfected with p20BAP31 for 48 h. **D** The relative quantification of the protein levels was analyzed by ImageJ software. *P < 0.05, **P < 0.01 and ***P < 0.001

The loss of MMP can lead to alterations in mitochondrial permeability and the release of proapoptotic proteins [23, 25]. It has been reported that p20BAP31 enhances the release of cytochrome c [16]. To further determine the mechanism of the p20BAP31-induced apoptosis pathway, we tested the expression of apoptosis-related proteins. The results showed that the cyt.c was released from the mitochondria to cytosol, the expression level of the antiapoptosis protein Bcl-2 was downregulated, while that of the proapoptotic protein Bax was elevated after transfected with p20BAP31 (Fig. 4A, B). It is generally believed that the elevated ratio of Bax and Bcl-2 leads to the activation of caspases. As shown in Fig. 4A, p20BAP31 significantly increased the production of cleaved caspase-9 and cleaved caspase-3. Caspase-3 is the most important terminal splicing enzyme in the process of cell apoptosis. The cleavage of caspase-3 is known to activate its proteolytic activity, which cleaves and thereby inactivates poly (ADP-ribose) polymerase (PARP) [48]. The results showed that p20BAP31 effectively increased the production of cleaved PARP (Fig. 4A). Considering the observed evidence, p20BAP31 could induce mitochondrial-dependent apoptosis in HCT116 cells.

### The MAPK signaling pathway is involved in p20BAP31‑induced apoptosis

The MAPK signaling pathway has been shown to play essential roles in regulating a variety of cellular processes, including cell growth, cell cycle regulation, migration, differentiation, development, and apoptosis [34]. To investigate the effect of p20BAP31 on the MAPK signaling pathway, we used western blot analysis to investigate the expression of p38, ERK, and JNK in HCT116 cells. As shown in Fig. 4C and D, compared with the control and mock groups, the phosphorylation levels of p38, ERK, and JNK were obviously increased in the cells transfected with p20BAP31. These results indicated that the MAPK signaling pathway is involved in the apoptosis induced by p20BAP31.

### p20BAP31-induced apoptosis is mediated via activation of the ROS-JNK pathway

**Fig. 5:**
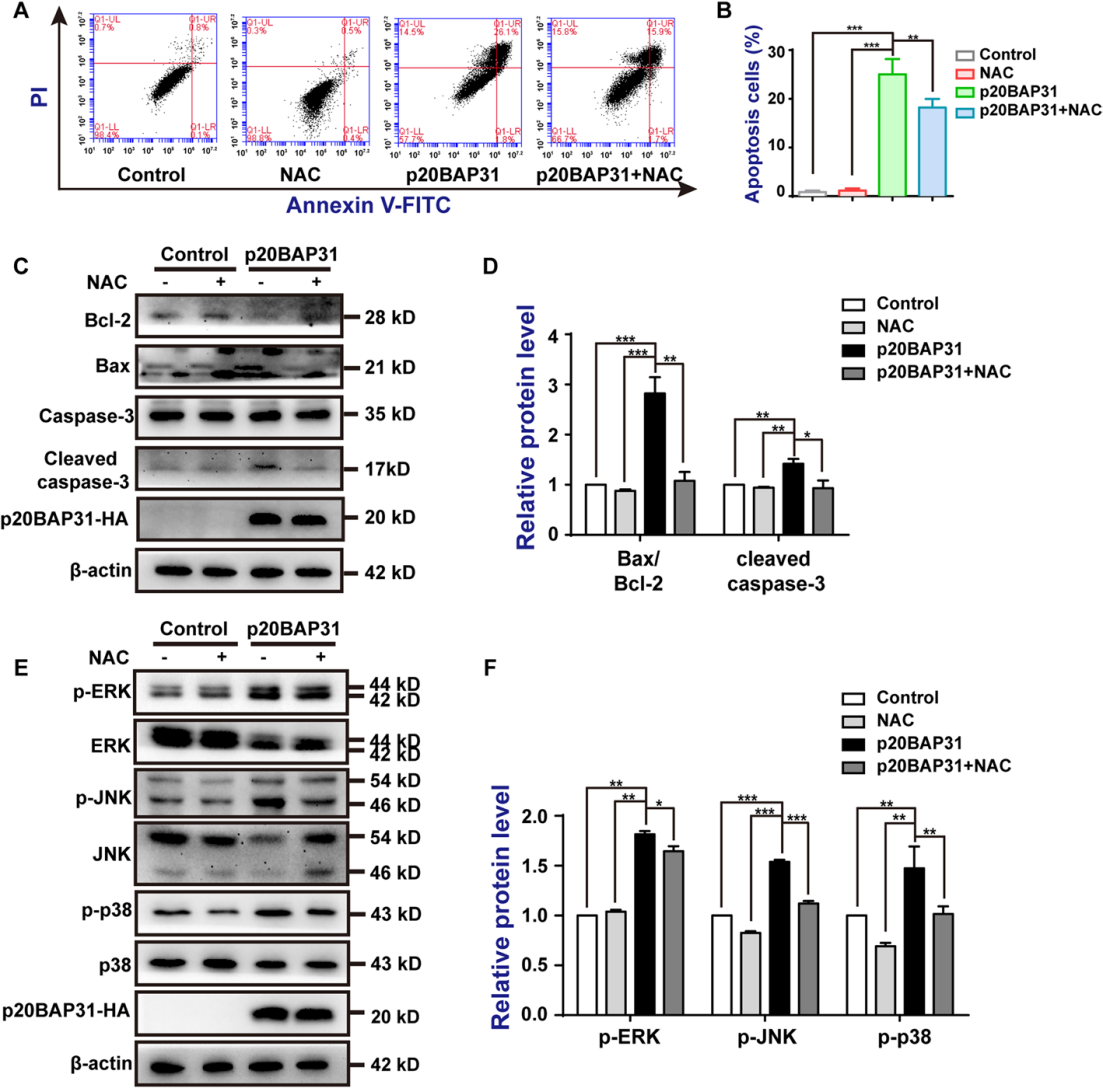
Roles of ROS generation in apoptosis induced by p20BAP31. Cells were transfected with p20BAP31 for 48 h, with or without pretreatment, with 5 mM NAC for 2 h. **A** Apoptosis of cells was assessed by flow cytometry analyses of annexin V-FITC/PI double-staining after treatment.** B** Quantitative analysis of apoptosis rates by flow cytometry.** C** The protein expression levels of Bcl-2, Bax, caspase-3, and cleaved caspase-3 in HCT116 cells were determined by western blotting. **D** Relative quantities analysis of Bax/Bcl-2 and cleaved caspase-3 in HCT116 cells. **E** Western blotting was used to detect p-ERK, ERK, p-JNK, JNK, p-p38, and p38 levels in HCT116 cells. **F** Relative quantities analysis of p-ERK, p-JNK, and p-p38 in HCT116 cells. *P < 0.05, **P < 0.01 and ***P < 0.001

ROS have been shown to participate in the regulation of apoptosis [49]. To further confirm the role of ROS in p20BAP31-induced cell apoptosis, the cells were pretreated with 5 mM NAC (a ROS scavenger). As expected, the green fluorescence intensity and the results of flow cytometry both showed that NAC significantly reduced the accumulation of intracellular ROS caused by p20BAP31 (Additional file 4: Fig. S4A-C). Furthermore, flow cytometry assay indicated that NAC significantly attenuated p20BAP31-induced apoptosis, while NAC itself had no effect on cell apoptosis (Fig. 5A, B). Western blot results showed that NAC could inhibit the p20BAP31-induced elevated ratio of Bax/Bcl-2 and the cleavage of caspase-3 to a great extent (Fig. 5C, D). In addition, NAC strongly blocked p20BAP31-induced JNK phosphorylation, partly inhibited p38 phosphorylation, and slightly blocked ERK phosphorylation (Fig. 5E, F). Taken together, these results demonstrated that p20BAP31-elicited ROS triggered the intrinsic mitochondrial pathway of apoptosis in HCT116 cells, which may be closely related to JNK activation.

**Fig. 6:**
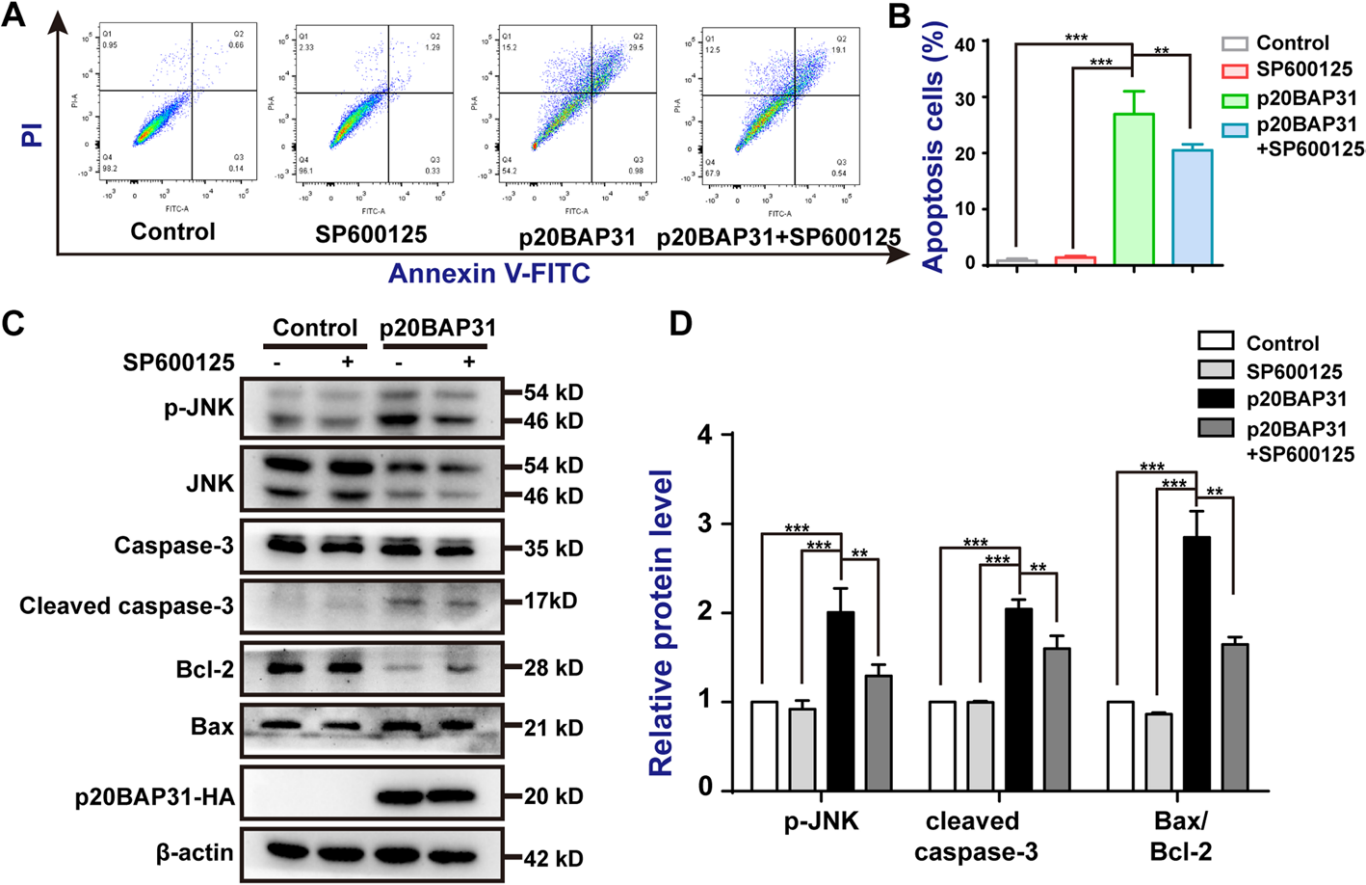
Roles of JNK in apoptosis induced by p20BAP31. Cells were transfected with p20BAP31 for 48 h, with or without pretreatment, with 10 μM SP600125 for 2 h. **A** Apoptosis of cells was assessed by flow cytometry analyses of annexin V-FITC/PI double-staining after treatment.** B** Quantitative analysis of apoptosis rates by flow cytometry.** C** The protein expression levels of p-JNK, caspase-3, cleaved caspase-3, Bcl-2, and Bax in HCT116 cells were determined by western blotting. **D** Relative quantities analysis of p-JNK, cleaved caspase-3, and Bax/Bcl-2 in HCT116 cells. **P < 0.01 and ***P < 0.001

To further determine the contribution of activated JNK to p20BAP31-induced apoptosis, we used the specific JNK inhibitor SP600125. Flow cytometry assays indicated that SP600125 attenuated p20BAP31-induced apoptosis (Fig. 6A, B). Western blot analysis showed that SP600125 inhibited p20BAP31-induced JNK phosphorylation and activation of apoptosis-related proteins to a certain extent (Fig. 6C, D). These results suggest that the activation of JNK is required for p20BAP31-induced apoptosis. Taken together, these results reveal that the ROS-JNK pathway is involved in p20BAP31-induced apoptosis.

### p20BAP31 induced both caspase-dependent and AIF-mediated caspase-independent apoptosis

**Fig. 7:**
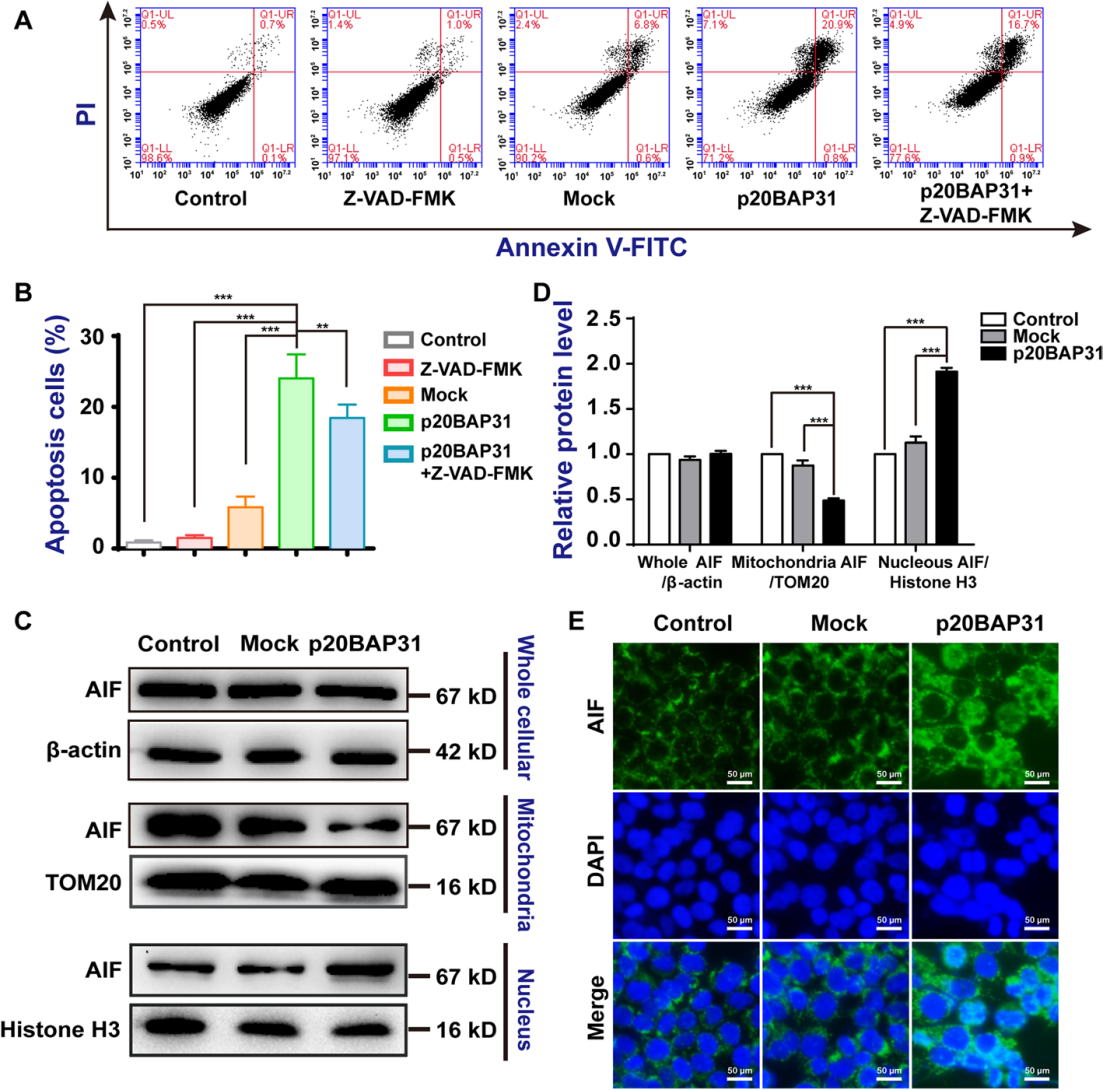
p20BAP31 induced AIF-mediated caspase-independent apoptosis.** A** HCT116 cells were pretreated with Z-VAD-FMK (20 μM) before transfection with p20BAP31 for 48 h, and the apoptosis rate was assessed by flow cytometry. **B** Quantitative analysis of apoptosis rates by flow cytometry. **C** Western blot analysis detected the protein expression of whole cellular, mitochondria, and nuclear AIF after transfection with p20BAP31 for 48 h. **D** The relative quantification of the protein levels was analyzed by ImageJ software. **E** Immunostaining of HCT116 cells was carried out with an anti-AIF antibody and a FITC secondary antibody (green) to localize AIF. Hoechst 33,342 staining (blue) was used to visualize the nuclei, and the merged images clearly demonstrate the nuclear localization of AIF. ***P < 0.001

Due to the production of ROS, the loss of MMP can result in caspase-independent cell death with subsequent release of mitochondrial apoptosis proteins such as apoptosis-inducing factor (AIF). As a caspase-independent apoptotic effector, AIF promotes cell death when it is released from the mitochondria and translocates into the nucleus to destroy cellular DNA, resulting in apoptosis [50, 51]. Moreover, AIF-induced apoptosis cannot be blocked by the general caspase inhibitor Z-VAD-FMK [52]. To investigate whether p20BAP31 induces caspase-independent apoptosis, we performed flow cytometry to test the apoptosis proportion of HCT116 cells treated with Z-VAD-FMK, the pan-caspase inhibitor, before transfection with p20BAP31. As shown in Fig. 7A and B, compared with the control and mock groups, the apoptosis proportion was significantly increased in the cells transfected with p20BAP31. Z-VAD-FMK, given alone, did not change the apoptosis proportion; however, pretreatment of HCT116 cells with Z-VAD-FMK only partly reversed the apoptosis proportion of p20BAP31 (Fig. 7A, B). These results showed that Z-VAD-FMK cannot completely block cell apoptosis, which means that p20BAP31 may induce caspase-independent cell apoptosis.

To further examine the role of AIF in apoptosis induced by p20BAP31, we extensively analyzed the protein levels of AIF by western blotting. The whole cellular AIF was basically unchanged after transfection with p20BAP31. The content of AIF in the mitochondria decreased, in accordance with the increased accumulation of AIF in the nuclei (Fig. 7C, D). Immunofluorescence staining of AIF in nuclei showed the same tendency (Fig. 7E). These results suggest that p20BAP31 triggers caspase-independent apoptosis that is dependent on the nuclear translocation of AIF.

## Discussion

**Fig. 8:**
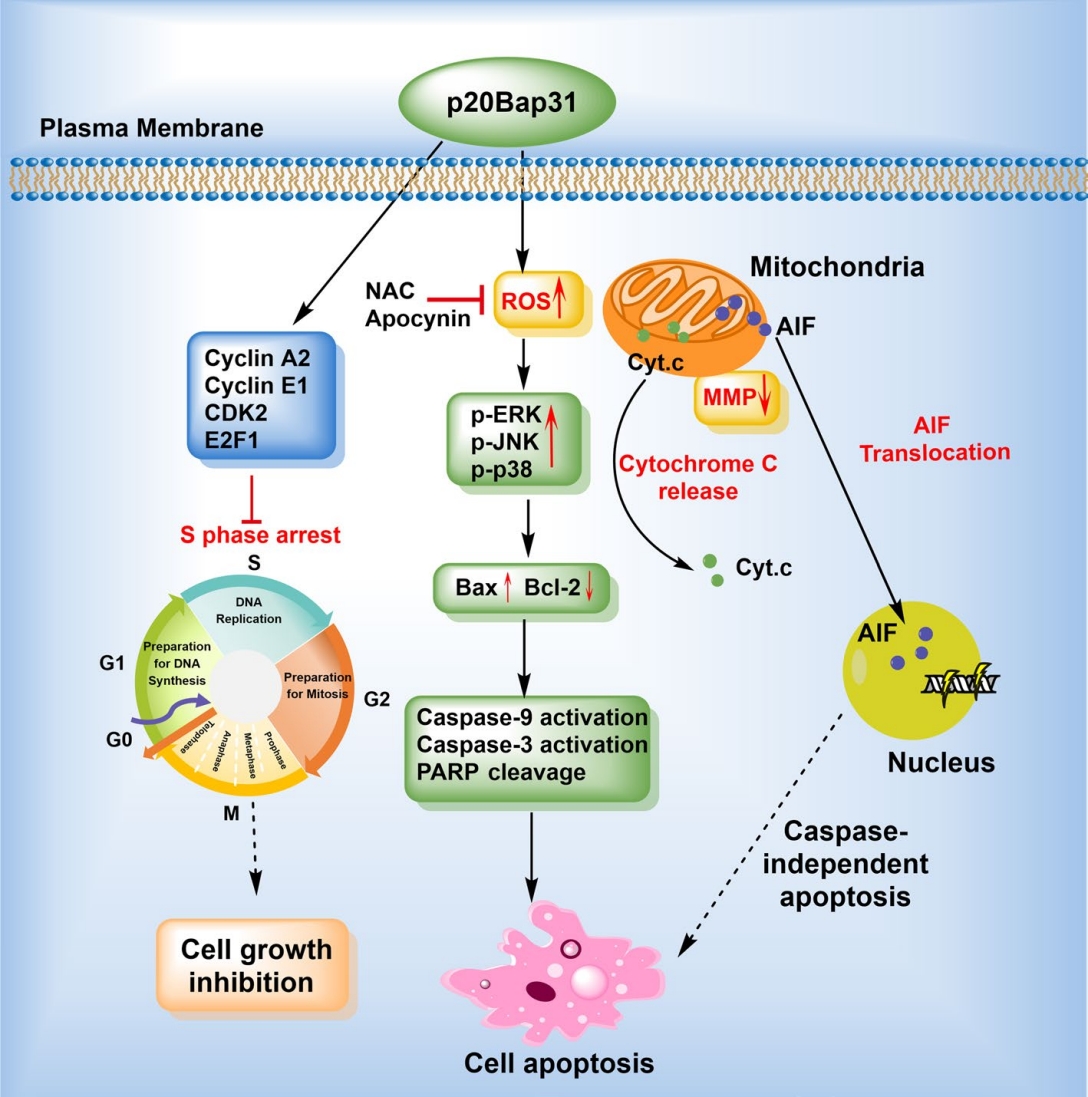
Schematic drawing of the proposed mechanisms of p20BAP31-induced apoptosis. See text for details

Previous studies have reported that p20BAP31 causes an early release of Ca^2+^ from the ER, leading to mitochondrial fission and the release of cyt.c [16], and initiates the Bax/Bak-independent paraptosis-like cell death pathway, which can be resisted by ER-localized Bcl-2 [18]. However, the underlying mechanisms of p20BAP31-induced cell apoptosis remain poorly understood. In the present study, we demonstrated that p20BAP31 significantly inhibited cell growth and induced S phase cell cycle arrest. A mechanistic study revealed that p20BAP31 induces apoptosis mediated by the ROS/JNK intrinsic mitochondrial pathway and the AIF-mediated caspase-independent pathway (Fig. 8).

To explore the mechanisms of p20BAP31-induced cell apoptosis, we first verified the effects of p20BAP31-induced apoptosis on various cell lines, and the results showed that the apoptosis rate of the HCT116 cells was highest in all the tested cells. Through Hoechst 33,342 staining, we clearly observed the obvious morphological features of apoptosis induced by p20BAP31 in HCT116 cells. Colorectal cancer (CRC) is one of the deadliest cancers worldwide [53], the mortality of which is the third highest among all cancers [54]; however, effective therapies remain limited. At present, targeting apoptosis is the most successful method to treat cancer, in addition to surgery. Therefore, it is of great significance to explore the potential mechanisms of apoptosis in HCT116 cells induced by p20BAP31.

The inhibition of cell survival and proliferation through apoptotic pathways is a promising therapeutic strategy for cancer therapy [55]. In our study, the CCK-8 assay and colony formation experiments revealed that p20BAP31 has a significant inhibitory effect on the proliferation of HCT116 cells. The common biological feature of tumors is uncontrolled growth, the main molecular mechanism of which is cell cycle disorder that leads to excessive cell proliferation and less apoptosis [56]. Our previous experiments have proven that p20BAP31 can induce apoptosis and inhibit cell proliferation; therefore, we further explored whether it is related to cell cycle disorders. Cell cycle analysis and western blot assays both showed that p20BAP31 induced S phase cycle arrest in HCT116 cells, which verified our hypothesis. In addition, apoptotic cells are recognized as having diminished DNA content (noted sub-G1) or morphologic changes [36], and our results showed that p20BAP31 could significantly increase the sub-G1 population. E2F transcription factor 1 (E2F1), one of the E2F family of transcription factors, is a key regulator of numerous genes involved in cell cycle progression [57]. Previous studies have shown that E2F1 regulates the activity of cyclin A2-CDK2 and cyclin E1-CDK2 complexes [39], and we found that p20BAP31 effectively reduced the protein levels of E2F1. These data indicate that p20BAP31 inhibits the proliferation of HCT116 cells via E2F1-regulated S phase cell cycle arrest. Furthermore, E2F transcription factors (E2Fs) can activate or silence many oncogenes or tumor suppressor genes in various malignancies, which have been shown to be involved in cell proliferation, differentiation, apoptosis, metastasis, and chemoresistance in colorectal cancer [58]. E2F1 has been found to promote the proliferation of cancer cells by transactivating cell cycle-related kinases. Therefore, we hypothesized that p20BAP31 induction of apoptosis was more sensitive in cancer cells, possibly because p20BAP31 inhibited the expression of E2F1, which in turn inhibits cancer cell proliferation by inhibiting the expression of tumor-related factors. The difference in the induction of apoptosis by p20BAP31 in cancer cells and normal cells will be further investigated in our future experiments.

The apoptosis pathway is widely divided into two major types: the intrinsic pathway (mediated by mitochondria) and the extrinsic pathway (mediated by death receptors) [59]. BAP31 itself is a target of regulation during the cellular response to death receptor stimulation [15, 60]. To identify the type of p20BAP31-induced apoptosis, we observed the change in intracellular mitochondrial membrane potential by JC-1. The results suggest that p20BAP31 caused the collapse of mitochondrial membrane potential. Then, we examined the expression levels of apoptosis-associated proteins. As we expected, p20BAP31 induced the release of cyt.c, inhibited the expression of the apoptosis protein Bcl-2, increased the expression of the apoptosis protein Bax, and further activated the downstream apoptosis-inducing caspase-9, caspase-3, and cleaved PARP, which eventually led to apoptosis. These results indicate that p20BAP31 could induce intrinsic mitochondrial pathway apoptosis.

Next, we explored the upstream pathways. Intracellular ROS participate in the regulation of cell apoptosis. Therefore, we first detected the production of ROS and found that p20BAP31 significantly increased intracellular ROS content. The ROS production of p20BAP31 may be mainly from NOX2. Moreover, the ROS inhibitor NAC significantly reduced p20BAP31-induced ROS production and apoptosis, suggesting that ROS generation is involved in the mechanism of p20BAP31-induced apoptosis in HCT116 cells. It is well-documented that excessive generation of ROS could interfere with various cellular signaling pathways. Mitogen-activated protein kinases (MAPKs) are a family of proteins that play essential roles in regulating many cellular processes [61]. The present study showed that overexpression of p20BAP31 resulted in the activation of ERK, JNK, and p38. Furthermore, we also found that NAC blocks the activation of the MAPK pathway and mainly inhibits the JNK phosphorylation induced by p20BAP31. Therefore, we further examined the effect of a JNK inhibitor (SP600125) on cell apoptosis induced by p20BAP31 and found that the JNK inhibitor significantly reversed p20BAP31-induced cell apoptosis, which revealed that JNK is critical in p20BAP31-induced apoptosis. In addition, JNK phosphorylation was potently abolished by NAC, implying that ROS are the proximal event of JNK. These data suggest that p20BAP31 induced apoptosis through the ROS/JNK signaling pathway.

Many studies have shown that high levels of ROS can change the mitochondrial permeability transition pores, leading to the translocation of AIF from mitochondria to the nucleus [62, 63]. It has been reported that AIF plays a central role in regulating caspase-independent pathways in cells [64]. We verified that p20BAP31 could induce changes in ROS content and mitochondrial membrane potential; therefore, we wanted to further identify whether p20BAP31 induces AIF-mediated apoptosis. We found that the pan-caspase inhibitor Z-VAD-FMK can partly block cell apoptosis induced by p20BAP31, which means that p20BAP31 may induce caspase-independent cell apoptosis. Western blotting and immunofluorescence assays both showed that AIF was released from the mitochondria to the nucleus. These results confirm that p20BAP31 also induces AIF-mediated caspase-independent apoptosis. The mechanisms of AIF-mediated apoptosis induced by p20BAP31 need to be further studied in the future.

In conclusion, the data presented herein suggested several aspects of the mechanisms of p20BAP31-induced apoptosis in HCT116 cells. A schematic drawing of the proposed mechanisms of p20BAP31-induced apoptosis is shown in Fig. 8. We propose that (i) p20BAP31 can effectively inhibit the proliferation of HCT116 cells via E2F1-regulated S phase cell cycle arrest, (ii) p20BAP31 induces intrinsic mitochondrial-dependent apoptosis by activating the ROS/JNK signaling pathway, and (iii) p20BAP31 induces AIF-mediated caspase-independent apoptosis by changing the mitochondrial permeability transition pores, leading to the translocation of AIF from mitochondria to the nucleus. These results provide valuable insights for the comprehensive study of p20BAP31-induced apoptosis and the in-depth exploration of its molecular mechanism.

## Conclusions

We demonstrated that p20BAP31 induced different levels of apoptosis in various cells and further examined the potential molecular mechanisms of apoptosis induced by p20BAP31. p20BAP31 induced mitochondrial-dependent apoptosis in HCT116 cells by activating the ROS/JNK signaling pathway, and induced S phase cell arrest and inhibited cell proliferation. Furthermore, p20BAP31 was also found for the first time to induce AIF-mediated apoptosis independent of caspase. Collectively, the results revealed that p20BAP31 could induce apoptosis through diverse pathways and may be a promising candidate for cancer therapy.

## Supplementary Information

**Additional file 1 Fig. S1** p20BAP31 induced cell apoptosis in various cells. (A), (B) The protein levels of p20BAP31 were measured by western blot after transfection with p20BAP31 for 48 h in different cells. (C) The transfection efficiency and cellular morphology of different cells were observed by fluorescence microscopy after transfection with p20BAP31 for 48 h. *P < 0.05.

**Additional file 2 Fig. S2** The transfection efficiency of overexpressed p20BAP31 at different times. The transfection efficiency and cellular morphology of HCT116 cells transfected with p20BAP31 at 12 h, 24 h, 48 h, and 72 h were observed by fluorescence microscopy.

**Additional file 3 Fig. S3** p20BAP31 induced cell cycle arrest at S phase. (A) Flow cytometry was used to determine the cell cycle distribution of HCT116 cells transfected with p20BAP31 for 24 h. (B) Statistical analysis of the cell cycle distribution of HCT116 cells transfected with p20BAP31 for 24 h. (C) Flow cytometry was used to determine the cell cycle distribution of HCT116 cells transfected with p20BAP31 for 36 h. (D) Statistical analysis of the cell cycle distribution of HCT116 cells transfected with p20BAP31 for 36 h.

**Additional file 4 Fig. S4** NAC inhibited ROS generation induced by p20BAP31. Cells were transfected with p20BAP31 for 48 h, with or without pretreatment of 5 mM NAC for 2 h. (A) Cells were collected and DCFH-DA fluorescence was observed by fluorescence microscope. (B) Flow cytometry was used to determine the alteration of ROS content in HCT116 cells. (C) Relative quantitative analysis of ROS in HCT116 cells. ***P < 0.001.

**Additional file 5 Table S1.** Sequences of primers for PCR.

## Data Availability

The data used to support the findings of this study are available from the corresponding authors upon request.

## Abbreviations

BAP31: B-cell receptor associated protein 31
ER: Endoplasmic reticulum
HCT116: Human colorectal carcinoma cell line
MMP: Mitochondrial membrane potential
ROS: Reactive oxygen species
MAPK: Mitogen-activated protein kinase
AIF: Apoptosis inducing factor
CRC: Colorectal cancer
hESC: Human embryonic stem cell
E2F1: E2F transcription factor 1
cyt.c: Cytochrome c
DN: Dominant negative
PCD: Programmed cell death
NAC: *N*-acetyl-l-cysteine
OD: Optical density
CCK-8: Cell counting kit-8
PI: Propidium iodide
ANOVA: Analysis of variance
CDKs: Cyclin-dependent kinases
PARP: Poly(ADP-ribose) polymerase

## References

[1] Adachi T, Schamel WW, Kim KM, Watanabe T, Becker B, Nielsen PJ (1996). The specificity of association of the IgD molecule with the accessory proteins BAP31/BAP29 lies in the IgD transmembrane sequence. EMBO J.

[2] Kim KM, Adachi T, Nielsen PJ, Terashima M, Lamers MC, Köhler G (1994). Two new proteins preferentially associated with membrane immunoglobulin D. EMBO J.

[3] Ng FW, Nguyen M, Kwan T, Branton PE, Nicholson DW, Cromlish JA (1997). p28 Bap31, a Bcl-2/Bcl-XL- and procaspase-8-associated protein in the endoplasmic reticulum. J Cell Biol.

[4] Szczesna-Skorupa E, Kemper B (2006). BAP31 is involved in the retention of cytochrome P450 2C2 in the endoplasmic reticulum. J Biol Chem.

[5] Annaert WG, Becker B, Kistner U, Reth M, Jahn R (1997). Export of cellubrevin from the endoplasmic reticulum is controlled by BAP31. J Cell Biol.

[6] Ladasky JJ, Boyle S, Seth M, Li H, Pentcheva T, Abe F (2006). Bap31 enhances the endoplasmic reticulum export and quality control of human class I MHC molecules. J Immunol.

[7] Zen K, Utech M, Liu Y, Soto I, Nusrat A, Parkos CA (2004). Association of BAP31 with CD11b/CD18. Potential role in intracellular trafficking of CD11b/CD18 in neutrophils. J Biol Chem.

[8] Wang B, Heath-Engel H, Zhang D, Nguyen N, Thomas DY, Hanrahan JW (2008). BAP31 interacts with Sec61 translocons and promotes retrotranslocation of CFTRDeltaF508 via the derlin-1 complex. Cell.

[9] Niu K, Xu J, Cao Y, Hou Y, Shan M, Wang Y (2017). BAP31 is involved in T cell activation through TCR signal pathways. Sci Rep.

[10] Xu J-L, Li L-Y, Wang Y-Q, Li Y-Q, Shan M, Sun S-Z (2018). Hepatocyte-specific deletion of BAP31 promotes SREBP1C activation, promotes hepatic lipid accumulation, and worsens IR in mice. J Lipid Res.

[11] Chen J, Guo H, Jiang H, Namusamba M, Wang C, Lan T (2019). A BAP31 intrabody induces gastric cancer cell death by inhibiting p27 proteasome degradation. Int J Cancer.

[12] Kim W-T, Seo Choi H, Min Lee H, Jang Y-J, Ryu CJ (2014). B-cell receptor-associated protein 31 regulates human embryonic stem cell adhesion, stemness, and survival via control of epithelial cell adhesion molecule. Stem Cells.

[13] Namba T (2019). BAP31 regulates mitochondrial function via interaction with Tom40 within ER-mitochondria contact sites. Sci Adv.

[14] Machihara K, Namba T (2019). BAP31 inhibits cell adaptation to ER stress conditions, negatively regulating autophagy induction by interaction with STX17. Cells.

[15] Nguyen M, Breckenridge DG, Ducret A, Shore GC (2000). Caspase-resistant BAP31 inhibits fas-mediated apoptotic membrane fragmentation and release of cytochrome c from mitochondria. Mol Cell Biol.

[16] Breckenridge DG, Stojanovic M, Marcellus RC, Shore GC (2003). Caspase cleavage product of BAP31 induces mitochondrial fission through endoplasmic reticulum calcium signals, enhancing cytochrome c release to the cytosol. J Cell Biol.

[17] Stojanovic M, Germain M, Nguyen M, Shore GC (2005). BAP31 and its caspase cleavage product regulate cell surface expression of tetraspanins and integrin-mediated cell survival. J Biol Chem.

[18] Heath-Engel HM, Wang B, Shore GC (2012). Bcl2 at the endoplasmic reticulum protects against a Bax/Bak-independent paraptosis-like cell death pathway initiated via p20Bap31. Biochim Biophys Acta.

[19] Fuchs Y, Steller H (2015). Live to die another way: modes of programmed cell death and the signals emanating from dying cells. Nat Rev Mol Cell Biol.

[20] Sperandio S, de Belle I, Bredesen DE (2000). An alternative, nonapoptotic form of programmed cell death. Proc Natl Acad Sci U S A.

[21] Elmore S (2007). Apoptosis: a review of programmed cell death. Toxicol Pathol.

[22] McGlorthan L, Paucarmayta A, Casablanca Y, Maxwell GL, Syed V (2021). Progesterone induces apoptosis by activation of caspase-8 and calcitriol via activation of caspase-9 pathways in ovarian and endometrial cancer cells in vitro. Apoptosis.

[23] Edlich F (2018). BCL-2 proteins and apoptosis: Recent insights and unknowns. Biochem Biophys Res Commun.

[24] Mohamed MS, Abdelhamid AO, Almutairi FM, Ali AG, Bishr MK (2018). Induction of apoptosis by pyrazolo[3,4-d]pyridazine derivative in lung cancer cells via disruption of Bcl-2/Bax expression balance. Bioorgan Med Chem.

[25] Lopez J, Tait SWG (2015). Mitochondrial apoptosis: killing cancer using the enemy within. Br J Cancer.

[26] Ye H, Cande C, Stephanou NC, Jiang S, Gurbuxani S, Larochette N (2002). DNA binding is required for the apoptogenic action of apoptosis inducing factor. Nat Struct Biol.

[27] Parrish JZ, Xue D (2003). Functional genomic analysis of apoptotic DNA degradation in *C. elegans*. Mol Cell.

[28] Susin SA, Lorenzo HK, Zamzami N, Marzo I, Snow BE, Brothers GM (1999). Molecular characterization of mitochondrial apoptosis-inducing factor. Nature.

[29] Park SY, Kim HY, Lee JH, Yoon KH, Chang MS, Park SK (2010). The age-dependent induction of apoptosis-inducing factor (AIF) in the human semitendinosus skeletal muscle. Cell Mol Biol Lett.

[30] Kang SW, Lee S, Lee EK (2015). ROS and energy metabolism in cancer cells: alliance for fast growth. Arch Pharm Res.

[31] Redza-Dutordoir M, Averill-Bates DA (2016). Activation of apoptosis signalling pathways by reactive oxygen species. Biochim Biophys Acta.

[32] Sylvester AL, Zhang DX, Ran S, Zinkevich NS (2022). Inhibiting NADPH oxidases to target vascular and other pathologies: an update on recent experimental and clinical studies. Biomolecules.

[33] Darling NJ, Cook SJ (2014). The role of MAPK signalling pathways in the response to endoplasmic reticulum stress. Biochim Biophys Acta.

[34] Zhang W, Liu HT (2002). MAPK signal pathways in the regulation of cell proliferation in mammalian cells. Cell Res.

[35] Quistgaard EM (2021). BAP31: physiological functions and roles in disease. Biochimie.

[36] Plesca D, Mazumder S, Almasan A (2008). Chapter 6 DNA damage response and apoptosis. Method Enzymol..

[37] Wu C-W, Storey KB (2012). Pattern of cellular quiescence over the hibernation cycle in liver of thirteen-lined ground squirrels. Cell Cycle.

[38] Kozar K, Ciemerych MA, Rebel VI, Shigematsu H, Zagozdzon A, Sicinska E (2004). Mouse development and cell proliferation in the absence of D-cyclins. Cell.

[39] Dapas B, Farra R, Grassi M, Giansante C, Fiotti N, Uxa L, Rainaldi G (2009). Role of E2F1-cyclin E1-cyclin E2 circuit in human coronary smooth muscle cell proliferation and therapeutic potential of its downregulation by siRNAs. Mol Med.

[40] Yao H, Lu F, Shao Y (2020). The E2F family as potential biomarkers and therapeutic targets in colon cancer. PeerJ.

[41] Bertoli C, Skotheim JM, de Bruin RAM (2013). Control of cell cycle transcription during G1 and S phases. Nat Rev Mol Cell Biol.

[42] Kroemer G, Galluzzi L, Brenner C (2007). Mitochondrial membrane permeabilization in cell death. Physiol Rev.

[43] Huang K-B, Wang F-Y, Tang X-M, Feng H-W, Chen Z-F, Liu Y-C (2018). Organometallic gold(III) complexes similar to tetrahydroisoquinoline induce ER-stress-mediated apoptosis and pro-death autophagy in A549 cancer cells. J Med Chem.

[44] Ren B, Li D, Si L, Ding Y, Han J, Chen X (2018). Alteronol induces cell cycle arrest and apoptosis via increased reactive oxygen species production in human breast cancer T47D cells. J Pharm Pharmacol.

[45] Marchi S, Giorgi C, Suski JM, Agnoletto C, Bononi A, Bonora M (2012). Mitochondria-ros crosstalk in the control of cell death and aging. J Signal Transduct.

[46] Pacelli C, Latorre D, Cocco T, Capuano F, Kukat C, Seibel P (2011). Tight control of mitochondrial membrane potential by cytochrome c oxidase. Mitochondrion.

[47] Yao C, Jiang J, Tu Y, Ye S, Du H, Zhang Y (2014). β-elemene reverses the drug resistance of A549/DDP lung cancer cells by activating intracellular redox system, decreasing mitochondrial membrane potential and P-glycoprotein expression, and inducing apoptosis. Thorac Cancer.

[48] Lyakhovich A, Surrallés J (2010). Constitutive activation of caspase-3 and Poly ADP ribose polymerase cleavage in fanconi anemia cells. Mol Cancer Res.

[49] Li HY, Zhang J, Sun LL, Li BH, Gao HL, Xie T (2015). Celastrol induces apoptosis and autophagy via the ROS/JNK signaling pathway in human osteosarcoma cells: an in vitro and in vivo study. Cell Death Dis.

[50] Bano D, Prehn JHM (2018). Apoptosis-inducing factor (AIF) in physiology and disease: the tale of a repented natural born killer. EBioMedicine.

[51] Lipton SA, Bossy-Wetzel E (2002). Dueling activities of AIF in cell death versus survival: DNA binding and redox activity. Cell.

[52] Joza N, Susin SA, Daugas E, Stanford WL, Cho SK, Li CY (2001). Essential role of the mitochondrial apoptosis-inducing factor in programmed cell death. Nature.

[53] Binefa G, Rodríguez-Moranta F, Teule A, Medina-Hayas M (2014). Colorectal cancer: from prevention to personalized medicine. World J Gastroenterol.

[54] Arnold M, Sierra MS, Laversanne M, Soerjomataram I, Jemal A, Bray F (2017). Global patterns and trends in colorectal cancer incidence and mortality. Gut.

[55] Carneiro BA, El-Deiry WS (2020). Targeting apoptosis in cancer therapy. Nat Rev Clin Oncol.

[56] Blagosklonny MV, Pardee AB (2001). Exploiting cancer cell cycling for selective protection of normal cells. Cancer Res.

[57] He Y, Huang S, Cheng T, Wang Y, Zhou SJ, Zhang YM (2020). High glucose may promote the proliferation and metastasis of hepatocellular carcinoma via E2F1/RRBP1 pathway. Life Sci.

[58] Fang Z, Lin M, Chen S, Liu H, Zhu M, Hu Y (2022). E2F1 promotes cell cycle progression by stabilizing spindle fiber in colorectal cancer cells. Cell Mol Biol Lett.

[59] Letai A (2017). Apoptosis and cancer. Annu Rev Cancer Biol.

[60] Wang B, Nguyen M, Breckenridge DG, Stojanovic M, Clemons PA, Kuppig S (2003). Uncleaved BAP31 in association with A4 protein at the endoplasmic reticulum is an inhibitor of Fas-initiated release of cytochrome c from mitochondria. J Biol Chem.

[61] Kostenko S, Dumitriu G, Lægreid KJ, Moens U (2011). Physiological roles of mitogen-activated-protein-kinase-activated p38-regulated/activated protein kinase. World J Biol Chem.

[62] Prasad N, Sharma JR, Yadav UCS (2020). Induction of growth cessation by acacetin via β-catenin pathway and apoptosis by apoptosis inducing factor activation in colorectal carcinoma cells. Mol Biol Rep.

[63] Schriewer JM, Peek CB, Bass J, Schumacker PT (2013). ROS-mediated PARP activity undermines mitochondrial function after permeability transition pore opening during myocardial ischemia-reperfusion. J Am Heart Assoc.

[64] Murahashi H, Azuma H, Zamzami N, Furuya K-J, Ikebuchi K, Yamaguchi M (2003). Possible contribution of apoptosis-inducing factor (AIF) and reactive oxygen species (ROS) to UVB-induced caspase-independent cell death in the T cell line Jurkat. J Leukoc Biol.

