# Supplementary figures and images for: p20BAP31 induces cell apoptosis via both AIF caspase-independent and the ROS/JNK mitochondrial pathway in colorectal cancer
Source: Cell Mol Biol Lett. 2023 Mar 28;28:25. doi: 10.1186/s11658-023-00434-z (PMC10052827; doi:10.1186/s11658-023-00434-z)

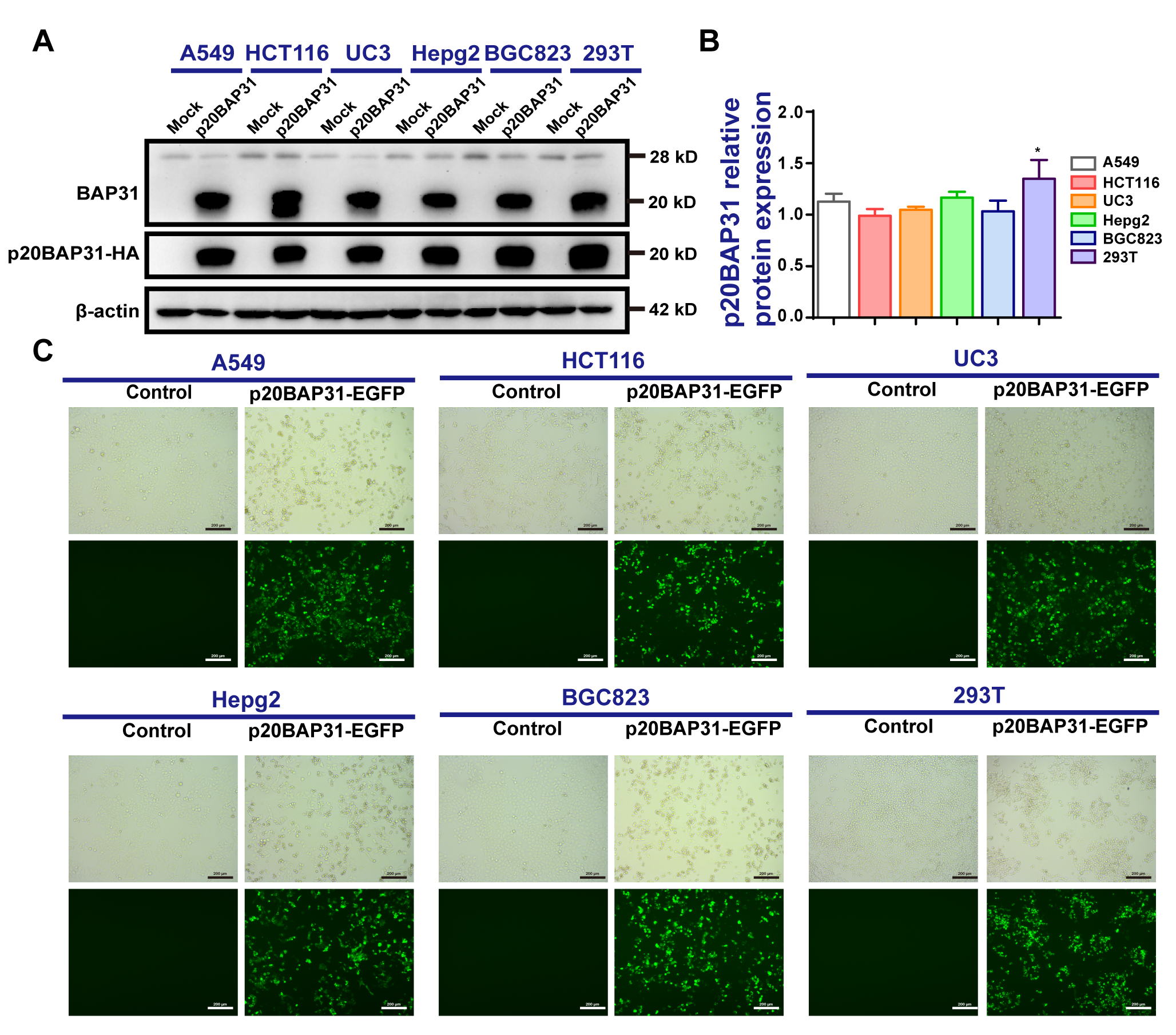

Supplement: Supplementary file 1 — Additional file 1 Fig. S1 p20BAP31 induced cell apoptosis in various cells. (A), (B) The protein levels of p20BAP31 were measured by western blot after transfection with p20BAP31 for 48 h in different cells. (C) The transfection efficiency and cellular morphology of different cells were observed by fluorescence microscopy after transfection with p20BAP31 for 48 h. *P < 0.05. [file 11658_2023_434_MOESM1_ESM.tif]

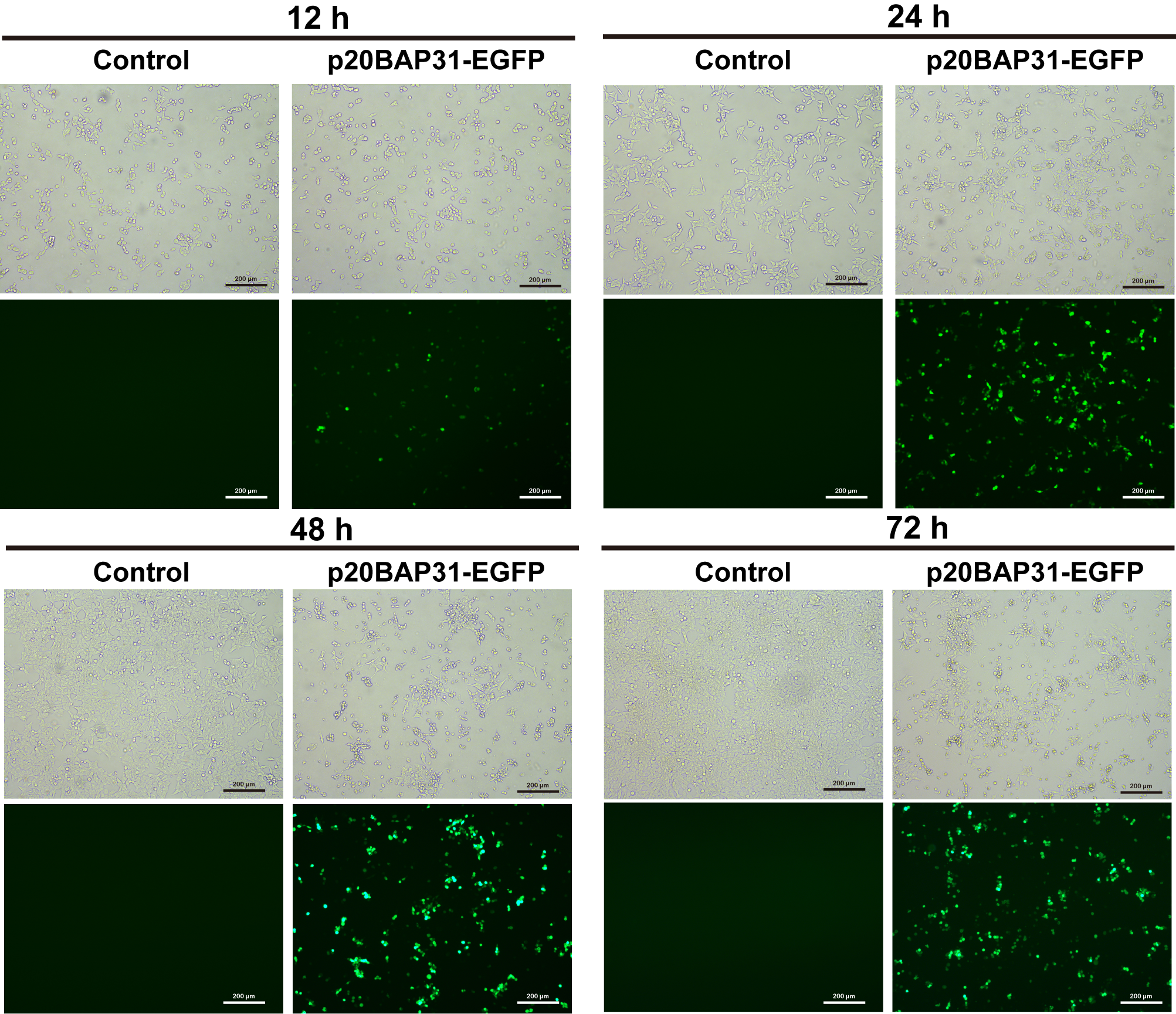

Supplement: Supplementary file 2 — Additional file 2 Fig. S2 The transfection efficiency of overexpressed p20BAP31 at different times. The transfection efficiency and cellular morphology of HCT116 cells transfected with p20BAP31 at 12 h, 24 h, 48 h, and 72 h were observed by fluorescence microscopy. [file 11658_2023_434_MOESM2_ESM.tif]

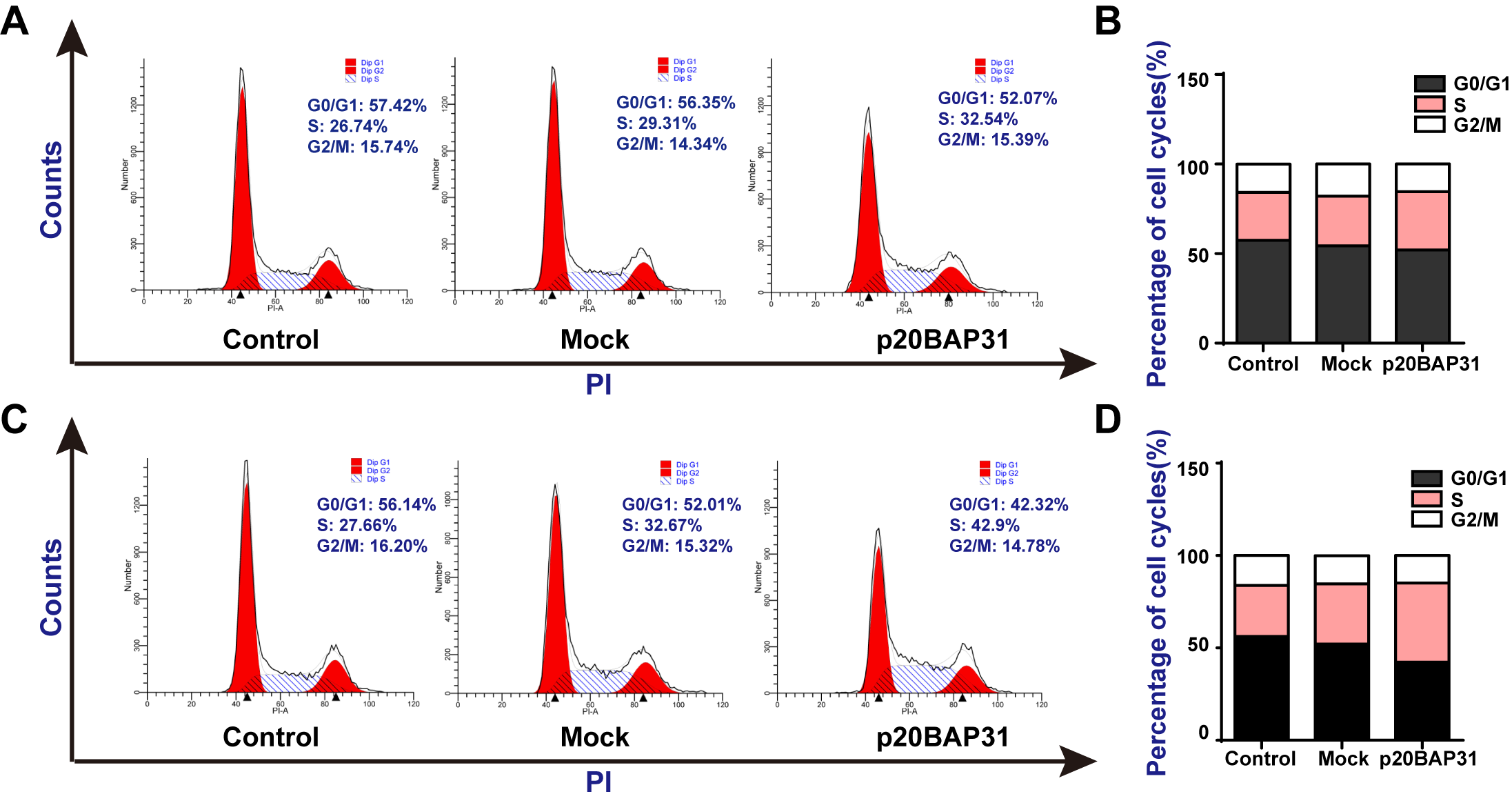

Supplement: Supplementary file 3 — Additional file 3 Fig. S3 p20BAP31 induced cell cycle arrest at S phase. (A) Flow cytometry was used to determine the cell cycle distribution of HCT116 cells transfected with p20BAP31 for 24 h. (B) Statistical analysis of the cell cycle distribution of HCT116 cells transfected with p20BAP31 for 24 h. (C) Flow cytometry was used to determine the cell cycle distribution of HCT116 cells transfected with p20BAP31 for 36 h. (D) Statistical analysis of the cell cycle distribution of HCT116 cells transfected with p20BAP31 for 36 h. [file 11658_2023_434_MOESM3_ESM.tif]

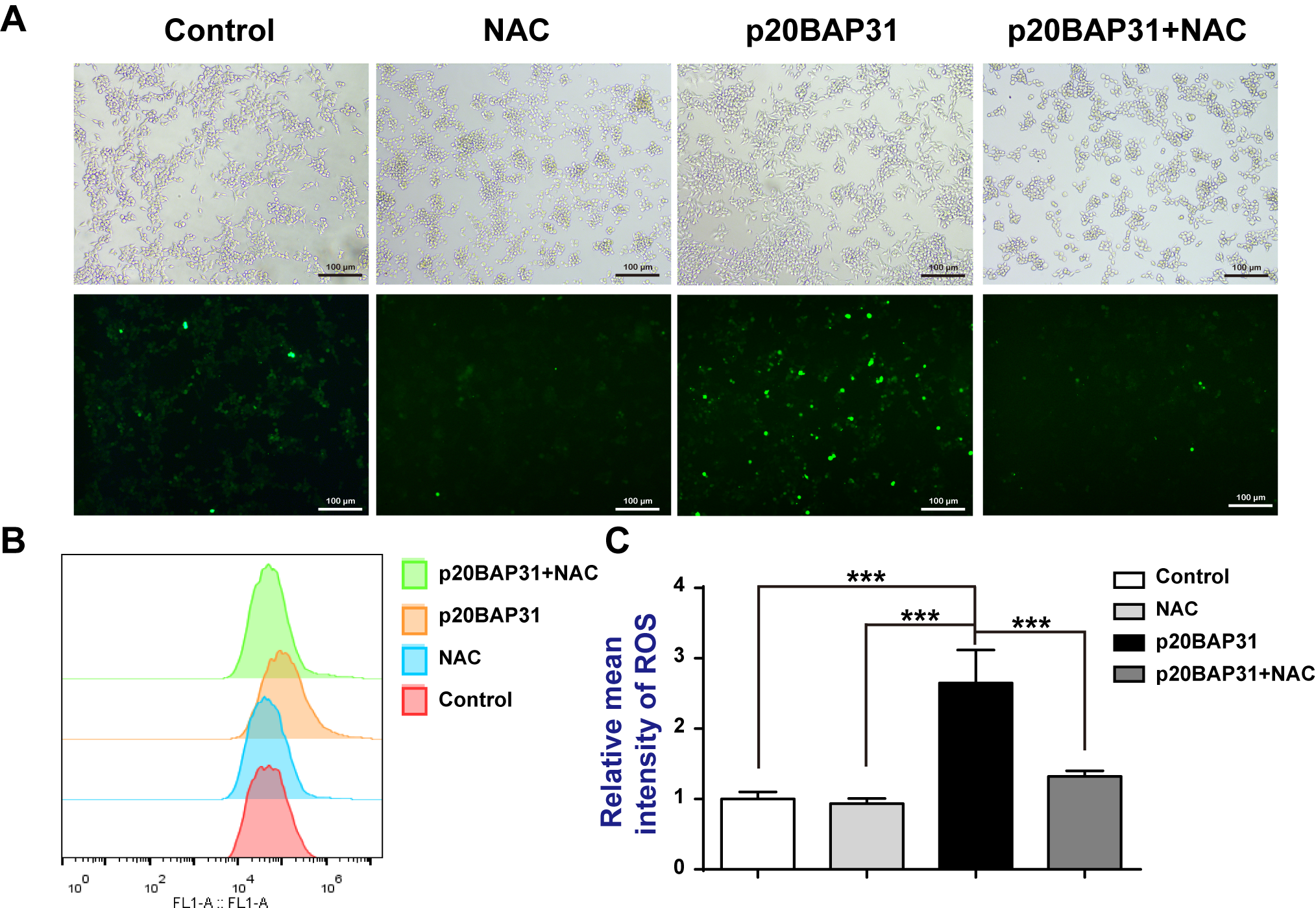

Supplement: Supplementary file 4 — Additional file 4 Fig. S4 NAC inhibited ROS generation induced by p20BAP31. Cells were transfected with p20BAP31 for 48 h, with or without pretreatment of 5 mM NAC for 2 h. (A) Cells were collected and DCFH-DA fluorescence was observed by fluorescence microscope. (B) Flow cytometry was used to determine the alteration of ROS content in HCT116 cells. (C) Relative quantitative analysis of ROS in HCT116 cells. ***P < 0.001. [file 11658_2023_434_MOESM4_ESM.tif]

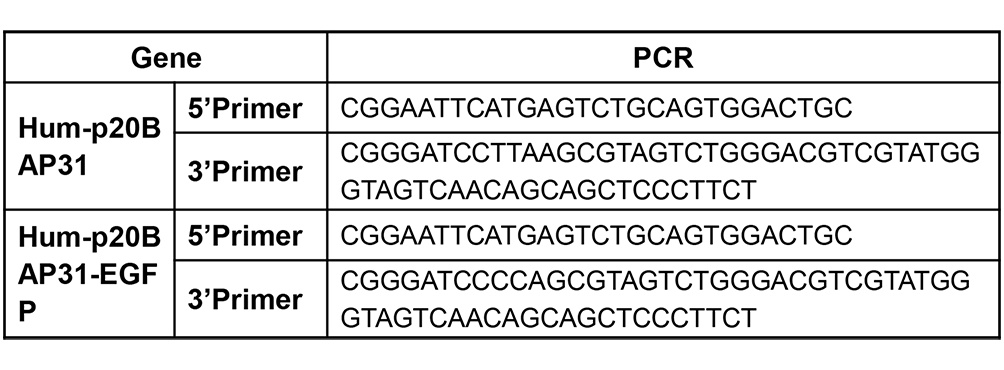

Supplement: Supplementary file 5 — Additional file 5 Table S1. Sequences of primers for PCR. [file 11658_2023_434_MOESM5_ESM.tif]
